# SARS-CoV-2 Omicron-B.1.1.529 leads to widespread escape from neutralizing antibody responses

**DOI:** 10.1016/j.cell.2021.12.046

**Published:** 2022-02-03

**Authors:** Wanwisa Dejnirattisai, Jiandong Huo, Daming Zhou, Jiří Zahradník, Piyada Supasa, Chang Liu, Helen M.E. Duyvesteyn, Helen M. Ginn, Alexander J. Mentzer, Aekkachai Tuekprakhon, Rungtiwa Nutalai, Beibei Wang, Aiste Dijokaite, Suman Khan, Ori Avinoam, Mohammad Bahar, Donal Skelly, Sandra Adele, Sile Ann Johnson, Ali Amini, Thomas G. Ritter, Chris Mason, Christina Dold, Daniel Pan, Sara Assadi, Adam Bellass, Nicola Omo-Dare, David Koeckerling, Amy Flaxman, Daniel Jenkin, Parvinder K. Aley, Merryn Voysey, Sue Ann Costa Clemens, Felipe Gomes Naveca, Valdinete Nascimento, Fernanda Nascimento, Cristiano Fernandes da Costa, Paola Cristina Resende, Alex Pauvolid-Correa, Marilda M. Siqueira, Vicky Baillie, Natali Serafin, Gaurav Kwatra, Kelly Da Silva, Shabir A. Madhi, Marta C. Nunes, Tariq Malik, Peter J.M. Openshaw, J. Kenneth Baillie, Malcolm G. Semple, Alain R. Townsend, Kuan-Ying A. Huang, Tiong Kit Tan, Miles W. Carroll, Paul Klenerman, Eleanor Barnes, Susanna J. Dunachie, Bede Constantinides, Hermione Webster, Derrick Crook, Andrew J. Pollard, Teresa Lambe, OPTIC Consortium, Christopher Conlon, Alexandra S. Deeks, John Frater, Lisa Frending, Siobhan Gardiner, Anni Jämsén, Katie Jeffery, Tom Malone, Eloise Phillips, Lucy Rothwell, Lizzie Stafford, ISARIC4C Consortium, J Kenneth Baillie, Malcolm G. Semple, Peter JM. Openshaw, Gail Carson, Beatrice Alex, Petros Andrikopoulos, Benjamin Bach, Wendy S. Barclay, Debby Bogaert, Meera Chand, Kanta Chechi, Graham S. Cooke, Ana da Silva Filipe, Thushan de Silva, Annemarie B. Docherty, Gonçalo dos Santos Correia, Marc-Emmanuel Dumas, Jake Dunning, Tom Fletcher, Christoper A. Green, William Greenhalf, Julian L. Griffin, Rishi K. Gupta, Ewen M. Harrison, Julian A. Hiscox, Antonia Ying Wai Ho, Peter W. Horby, Samreen Ijaz, Saye Khoo, Paul Klenerman, Andrew Law, Matthew R. Lewis, Sonia Liggi, Wei Shen Lim, Lynn Maslen, Alexander J. Mentzer, Laura Merson, Alison M. Meynert, Shona C. Moore, Mahdad Noursadeghi, Michael Olanipekun, Anthonia Osagie, Massimo Palmarini, Carlo Palmieri, William A. Paxton, Georgios Pollakis, Nicholas Price, Andrew Rambaut, David L. Robertson, Clark D. Russell, Vanessa Sancho-Shimizu, Caroline J. Sands, Janet T. Scott, Louise Sigfrid, Tom Solomon, Shiranee Sriskandan, David Stuart, Charlotte Summers, Olivia V. Swann, Zoltan Takats, Panteleimon Takis, Richard S. Tedder, AA Roger Thompson, Emma C. Thomson, Ryan S. Thwaites, Lance CW. Turtle, Maria Zambon, Hayley Hardwick, Chloe Donohue, Fiona Griffiths, Wilna Oosthuyzen, Cara Donegan, Rebecca G. Spencer, Lisa Norman, Riinu Pius, Thomas M. Drake, Cameron J. Fairfield, Stephen R. Knight, Kenneth A. Mclean, Derek Murphy, Catherine A. Shaw, Jo Dalton, Michelle Girvan, Egle Saviciute, Stephanie Roberts, Janet Harrison, Laura Marsh, Marie Connor, Sophie Halpin, Clare Jackson, Carrol Gamble, Daniel Plotkin, James Lee, Gary Leeming, Andrew Law, Murray Wham, Sara Clohisey, Ross Hendry, James Scott-Brown, Victoria Shaw, Sarah E. McDonald, Seán Keating, Katie A. Ahmed, Jane A. Armstrong, Milton Ashworth, Innocent G. Asiimwe, Siddharth Bakshi, Samantha L. Barlow, Laura Booth, Benjamin Brennan, Katie Bullock, Benjamin WA. Catterall, Jordan J. Clark, Emily A. Clarke, Sarah Cole, Louise Cooper, Helen Cox, Christopher Davis, Oslem Dincarslan, Chris Dunn, Philip Dyer, Angela Elliott, Anthony Evans, Lorna Finch, Lewis WS. Fisher, Terry Foster, Isabel Garcia-Dorival, Philip Gunning, Catherine Hartley, Rebecca L. Jensen, Christopher B. Jones, Trevor R. Jones, Shadia Khandaker, Katharine King, Robyn T. Kiy, Chrysa Koukorava, Annette Lake, Suzannah Lant, Diane Latawiec, Lara Lavelle-Langham, Daniella Lefteri, Lauren Lett, Lucia A. Livoti, Maria Mancini, Sarah McDonald, Laurence McEvoy, John McLauchlan, Soeren Metelmann, Nahida S. Miah, Joanna Middleton, Joyce Mitchell, Shona C. Moore, Ellen G. Murphy, Rebekah Penrice-Randal, Jack Pilgrim, Tessa Prince, Will Reynolds, P. Matthew Ridley, Debby Sales, Victoria E. Shaw, Rebecca K. Shears, Benjamin Small, Krishanthi S. Subramaniam, Agnieska Szemiel, Aislynn Taggart, Jolanta Tanianis-Hughes, Jordan Thomas, Erwan Trochu, Libby van Tonder, Eve Wilcock, J. Eunice Zhang, Lisa Flaherty, Nicole Maziere, Emily Cass, Alejandra Doce Carracedo, Nicola Carlucci, Anthony Holmes, Hannah Massey, Lee Murphy, Sarah McCafferty, Richard Clark, Angie Fawkes, Kirstie Morrice, Alan Maclean, Nicola Wrobel, Lorna Donnelly, Audrey Coutts, Katarzyna Hafezi, Louise MacGillivray, Tammy Gilchrist, Kayode Adeniji, Daniel Agranoff, Ken Agwuh, Dhiraj Ail, Erin L. Aldera, Ana Alegria, Sam Allen, Brian Angus, Abdul Ashish, Dougal Atkinson, Shahedal Bari, Gavin Barlow, Stella Barnass, Nicholas Barrett, Christopher Bassford, Sneha Basude, David Baxter, Michael Beadsworth, Jolanta Bernatoniene, John Berridge, Colin Berry, Nicola Best, Pieter Bothma, David Chadwick, Robin Brittain-Long, Naomi Bulteel, Tom Burden, Andrew Burtenshaw, Vikki Caruth, David Chadwick, Duncan Chambler, Nigel Chee, Jenny Child, Srikanth Chukkambotla, Tom Clark, Paul Collini, Catherine Cosgrove, Jason Cupitt, Maria-Teresa Cutino-Moguel, Paul Dark, Chris Dawson, Samir Dervisevic, Phil Donnison, Sam Douthwaite, Andrew Drummond, Ingrid DuRand, Ahilanadan Dushianthan, Tristan Dyer, Cariad Evans, Chi Eziefula, Chrisopher Fegan, Adam Finn, Duncan Fullerton, Sanjeev Garg, Sanjeev Garg, Atul Garg, Effrossyni Gkrania-Klotsas, Jo Godden, Arthur Goldsmith, Clive Graham, Elaine Hardy, Stuart Hartshorn, Daniel Harvey, Peter Havalda, Daniel B. Hawcutt, Maria Hobrok, Luke Hodgson, Anil Hormis, Michael Jacobs, Susan Jain, Paul Jennings, Agilan Kaliappan, Vidya Kasipandian, Stephen Kegg, Michael Kelsey, Jason Kendall, Caroline Kerrison, Ian Kerslake, Oliver Koch, Gouri Koduri, George Koshy, Shondipon Laha, Steven Laird, Susan Larkin, Tamas Leiner, Patrick Lillie, James Limb, Vanessa Linnett, Jeff Little, Mark Lyttle, Michael MacMahon, Emily MacNaughton, Ravish Mankregod, Huw Masson, Elijah Matovu, Katherine McCullough, Ruth McEwen, Manjula Meda, Gary Mills, Jane Minton, Mariyam Mirfenderesky, Kavya Mohandas, Quen Mok, James Moon, Elinoor Moore, Patrick Morgan, Craig Morris, Katherine Mortimore, Samuel Moses, Mbiye Mpenge, Rohinton Mulla, Michael Murphy, Megan Nagel, Thapas Nagarajan, Mark Nelson, Lillian Norris, Matthew K. O’Shea, Igor Otahal, Marlies Ostermann, Mark Pais, Carlo Palmieri, Selva Panchatsharam, Danai Papakonstantinou, Hassan Paraiso, Brij Patel, Natalie Pattison, Justin Pepperell, Mark Peters, Mandeep Phull, Stefania Pintus, Jagtur Singh Pooni, Tim Planche, Frank Post, David Price, Rachel Prout, Nikolas Rae, Henrik Reschreiter, Tim Reynolds, Neil Richardson, Mark Roberts, Devender Roberts, Alistair Rose, Guy Rousseau, Bobby Ruge, Brendan Ryan, Taranprit Saluja, Matthias L. Schmid, Aarti Shah, Prad Shanmuga, Anil Sharma, Anna Shawcross, Jeremy Sizer, Manu Shankar-Hari, Richard Smith, Catherine Snelson, Nick Spittle, Nikki Staines, Tom Stambach, Richard Stewart, Pradeep Subudhi, Tamas Szakmany, Kate Tatham, Jo Thomas, Chris Thompson, Robert Thompson, Ascanio Tridente, Darell Tupper-Carey, Mary Twagira, Nick Vallotton, Rama Vancheeswaran, Lisa Vincent-Smith, Shico Visuvanathan, Alan Vuylsteke, Sam Waddy, Rachel Wake, Andrew Walden, Ingeborg Welters, Tony Whitehouse, Paul Whittaker, Ashley Whittington, Padmasayee Papineni, Meme Wijesinghe, Martin Williams, Lawrence Wilson, Sarah Cole, Stephen Winchester, Martin Wiselka, Adam Wolverson, Daniel G. Wootton, Andrew Workman, Bryan Yates, Peter Young, Neil G. Paterson, Mark A. Williams, David R. Hall, Elizabeth E. Fry, Juthathip Mongkolsapaya, Jingshan Ren, Gideon Schreiber, David I. Stuart, Gavin R. Screaton

**Affiliations:** 1 Wellcome Centre for Human Genetics, Nuffield Department of Medicine, University of Oxford, Oxford, UK; 2 Division of Structural Biology, Nuffield Department of Medicine, University of Oxford, The Wellcome Centre for Human Genetics, Oxford, UK; 3 Chinese Academy of Medical Science (CAMS) Oxford Institute (COI), University of Oxford, Oxford, UK; 4 Department of Biomolecular Sciences, Weizmann Institute of Science, Rehovot, Israel; 5 Diamond Light Source Ltd, Harwell Science & Innovation Campus, Didcot, UK; 6 Oxford University Hospitals NHS Foundation Trust, Oxford, UK; 7 Peter Medawar Building for Pathogen Research, Oxford, UK; 8 Nuffield Department of Clinical Neurosciences, University of Oxford, Oxford, UK; 9 Translational Gastroenterology Unit, University of Oxford, Oxford, UK; 10 NIHR Oxford Biomedical Research Centre, Oxford, UK; 11 Oxford Vaccine Group, Department of Paediatrics, University of Oxford, Oxford, UK; 12 Department of Infectious Diseases and HIV Medicine, University Hospitals of Leicester NHS Trust, Leicester, UK; 13 Department of Respiratory Sciences, University of Leicester, Leicester, UK; 14 Medical Sciences Division, University of Oxford, Oxford, UK; 15 Jenner Institute, Nuffield Department of Medicine, University of Oxford, Oxford, UK; 16 Institute of Global Health, University of Siena, Siena, Brazil; 17 Laboratório de Ecologia de Doenças Transmissíveis na Amazônia, Instituto Leônidas e Maria Deane, Fiocruz, Manaus, Amazonas, Brazil; 18 Fundação de Vigilância em Saúde do Amazonas, Manaus, Amazonas, Brazil; 19 Laboratorio de vírus respiratórios-IOC/FIOCRUZ, Rio de Janeiro, Brazil; 20 Department of Veterinary Integrative Biosciences, Texas A&M University, College Station, TX, USA; 21 South African Medical Research Council, Vaccines and Infectious Diseases Analytics Research Unit, School of Pathology, Faculty of Health Sciences, University of the Witwatersrand, Johannesburg, South Africa; 22 Department of Science and Technology, National Research Foundation, South African Research Chair Initiative in Vaccine Preventable Diseases, Faculty of Health Sciences, University of the Witwatersrand, Johannesburg, South Africa; 23 National Infection Service, Public Health England (PHE), Porton Down, Salisbury, UK; 24 National Heart & Lung Institute, Imperial College, London, UK; 25 Genetics and Genomics, Roslin Institute, University of Edinburgh, Edinburgh, UK; 26 NIHR Health Protection Research Unit, Institute of Infection, Veterinary and Ecological Sciences, Faculty of Health and Life Sciences, University of Liverpool, Liverpool, UK; 27 MRC Human Immunology Unit, Weatherall Institute of Molecular Medicine, University of Oxford, John Radcliffe Hospital, Oxford, UK; 28 Division of Pediatric Infectious Diseases, Department of Pediatrics, Chang Gung Memorial Hospital and Research Center for Emerging Viral Infections, College of Medicine, Chang Gung University, Taoyuan, Taiwan; 29 Centre for Tropical Medicine and Global Health, Nuffield Department of Medicine, University of Oxford, Oxford, UK; 30 Mahidol-Oxford Tropical Medicine Research Unit, Bangkok, Thailand; 31 Nuffield Department of Medicine, University of Oxford, Oxford, UK; 32 Siriraj Center of Research Excellence in Dengue & Emerging Pathogens, Dean Office for Research, Faculty of Medicine Siriraj Hospital, Mahidol University, Bangkok, Thailand; 33 Instruct-ERIC, Oxford House, Parkway Court, John Smith Drive, Oxford, UK; 34 Department of Paediatrics, University of Oxford, Oxford, UK

**Keywords:** SARS-CoV-2, Omicron, variants, vaccines, immune evasion, receptor interaction, Spike, RBD

## Abstract

On 24^th^ November 2021, the sequence of a new SARS-CoV-2 viral isolate Omicron-B.1.1.529 was announced, containing far more mutations in Spike (S) than previously reported variants. Neutralization titers of Omicron by sera from vaccinees and convalescent subjects infected with early pandemic Alpha, Beta, Gamma, or Delta are substantially reduced, or the sera failed to neutralize. Titers against Omicron are boosted by third vaccine doses and are high in both vaccinated individuals and those infected by Delta. Mutations in Omicron knock out or substantially reduce neutralization by most of the large panel of potent monoclonal antibodies and antibodies under commercial development. Omicron S has structural changes from earlier viruses and uses mutations that confer tight binding to ACE2 to unleash evolution driven by immune escape. This leads to a large number of mutations in the ACE2 binding site and rebalances receptor affinity to that of earlier pandemic viruses.

## Highlights

• Large reduction in Omicron neutralization titers, ameliorated by the 3^rd^ booster vaccine
• Failure of many potent mAbs to neutralize Omicron
• Complex pattern of mutations balances ACE2 binding and antibody escape
• Omicron RBD is structurally similar but the most antigenically distant variant

## Graphical abstract

**Figure:**
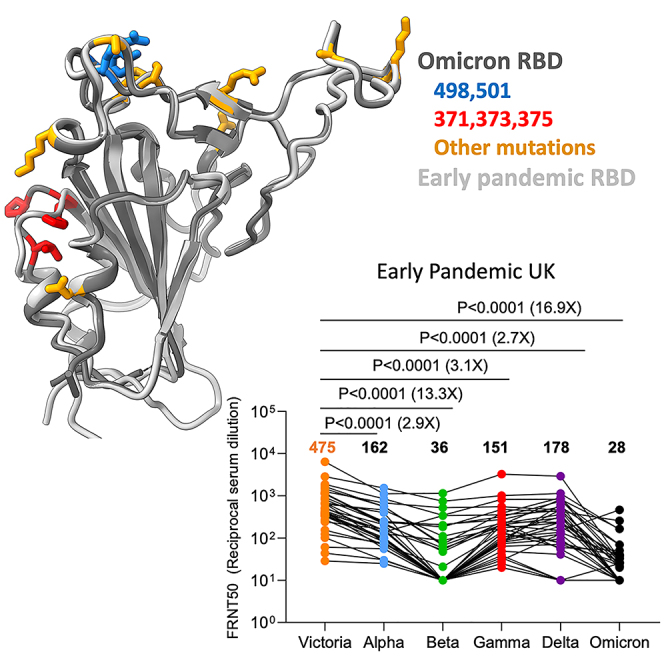

## Teaser

A comprehensive analysis of sera from vaccinees, convalescent patients previously infected by multiple variants, and potent monoclonal antibodies from early in the COVID-19 pandemic reveals a substantial overall reduction in the ability to neutralize the SARS-CoV-2 Omicron variant, which seems to ameliorate with a third vaccine dose. Structural analyses of the Omicron RBD suggest that selective pressure balances key changes that increase affinity for ACE2 with other changes in the receptor-binding motif that disfavor ACE2 binding but facilitate immune escape.

## Introduction

Since the end of 2020, a series of viral variants have been emerging in different regions, and some have caused large outbreaks. Alpha (Supasa et al., 2021) and, more recently, Delta (Liu et al., 2021a), have had the greatest global reach, whilst Beta (Zhou et al., 2021), Gamma (Dejnirattisai et al., 2021b), and Lambda (Colmenares-Mejía et al., 2021), despite causing large outbreaks in Southern Africa and South America, did not become dominant in other parts of the world. Indeed, Beta was later displaced by Delta in South Africa.

The rapid emergence of Omicron (https://www.who.int/news/item/26-11-2021-classification-of-omicron-(b.1.1.529)-sars-cov-2-variant-of-concern) in the background of high Beta immunity implies that the virus may have evolved to escape neutralization by Beta-specific serum (Liu et al., 2021b). Within Spike (S), Omicron has 30 substitutions plus a deletion of 6 and an insertion of 3 residues, whereas in all the other proteins there are a total of 16 substitutions and 7 residue deletions. Particular hotspots for the mutations are the angiotensin converting enzyme 2 (ACE2) receptor binding domain (RBD) (15 amino acid substitutions) and the N-terminal domain (NTD) (3 deletions totaling 6 residues, 1 insertion, and 4 substitutions).

S mediates cellular interactions. It is a dynamic, trimeric structure (Walls et al., 2017, 2020; Wrapp et al., 2020), which can be lipid bound (Toelzer et al., 2020) and tightly associated in a “closed” form or unfurled to expose one or more RBDs, allowing both receptor binding and increased access to neutralizing antibodies. Once bound to a cell, S undergoes cleavage and a drastic elongation, converting it to the post-fusion form.

Most potent neutralizing antibodies target the ACE2 footprint (Dejnirattisai et al., 2021a; Lan et al., 2020; Liu et al., 2021b), occupying ∼880 Å^2^ at the outermost tip of the RBD (the neck and shoulders, referring to the torso analogy [Dejnirattisai et al., 2021a]) and preventing cell attachment. A proportion of antibodies are able to cross-neutralize different variants (Liu et al., 2021b), and a few of these bind to a motif surrounding the N-linked glycan at residue 343 (Dejnirattisai et al., 2021a; Liu et al., 2021b). These latter antibodies, exemplified by S309 (Pinto et al., 2020), can cross-react with SARS-CoV-1 but do not block ACE2 interaction, and destabilizing the S-trimer may be their mechanism of action. Neutralizing anti-NTD mAbs do not block ACE2 interaction and bind to a so-called supersite on the NTD (Cerutti et al., 2021; Chi et al., 2020); however, they generally fail to provide a broad protection as the supersite is disrupted by a variety of NTD mutations present in the variants of concern (VOC). Moreover, some NTD-binding antibodies were shown to have an infectivity-enhancing effect by inducing the open form of S (Liu et al., 2021c).

In this report, we study the neutralization of Omicron by a large panel of sera collected from convalescents of early pandemic, Alpha-, Beta-, Gamma-, and Delta-infected individuals, together with vaccinees who had received three doses of the Oxford/AstraZeneca (AZD1222) or the Pfizer BioNtech (BNT16b2) vaccines. There is a widespread reduction in the neutralization activity of sera from multiple sources, and we use these data to plot an antigenic map, where Omicron is seen to occupy the most distant position from early pandemic viruses, which form the basis for current vaccines.

We show that Omicron escapes neutralization by the majority of potent monoclonal antibodies (mAbs) arising after both early pandemic and infection with Beta variant. Utilizing a large bank of structures (n = 29) from panels of potent mAbs, which includes most of the mAbs developed for prophylactic or therapeutic use, we describe the mechanism of escape caused by the numerous mutations present in Omicron RBD (Baum et al., 2020).

Analysis of the binding of ACE2 to RBD and structural analysis of the Omicron RBD indicate that changes at residues 498 and 501 of the RBD have locked ACE2 binding to the RBD in that region sufficiently strongly to enable the generation of a plethora of less favorable changes elsewhere, providing extensive immune escape and, in the process, resulting in a final net affinity for ACE2 similar to the early pandemic virus.

## Results

### Phylogeny of Omicron

**Figure 1:**
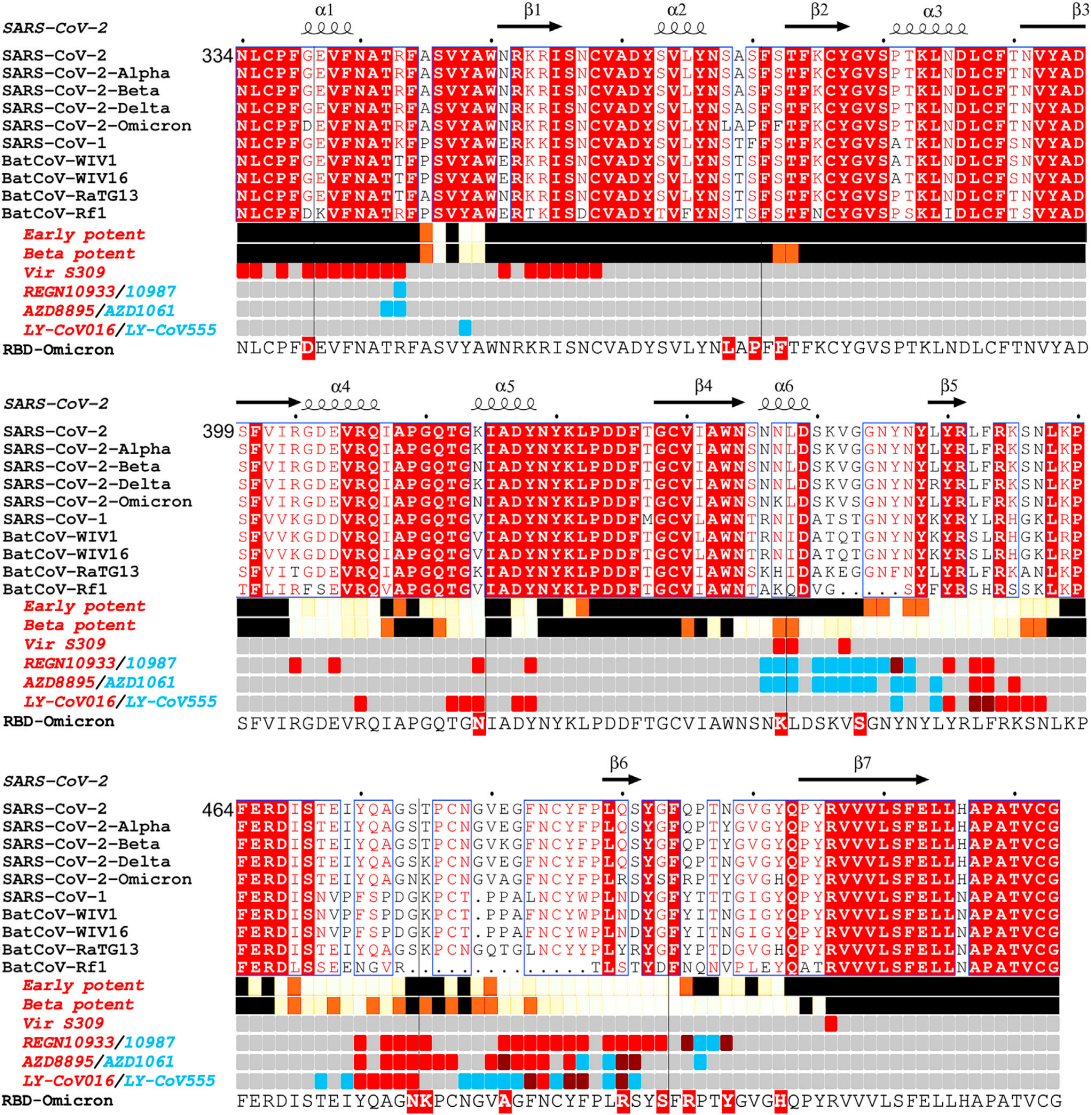
Sarbecovirus RBD sequence analysis Shown with Alpha, Beta, Delta, and Omicron variants (Omicron repeated on the lower line for clarity). Binding sites for the early pandemic potent antibodies (Dejnirattisai et al., 2021a) and the potent Beta antibodies (Liu et al., 2021b) are depicted using iron heat colors (black < straw < yellow < white) to indicate relative levels of antibody contact and commercial antibody contacts are depicted with the pairs of antibodies in red and blue (purple denotes common interactions). Totally conserved residues are boxed on a red background on the upper rows, while on the final row Omicron mutations are boxed in red. Secondary elements are denoted above the alignment. Figure produced in part using ESPript (Robert and Gouet, 2014).

**Figure 2:**
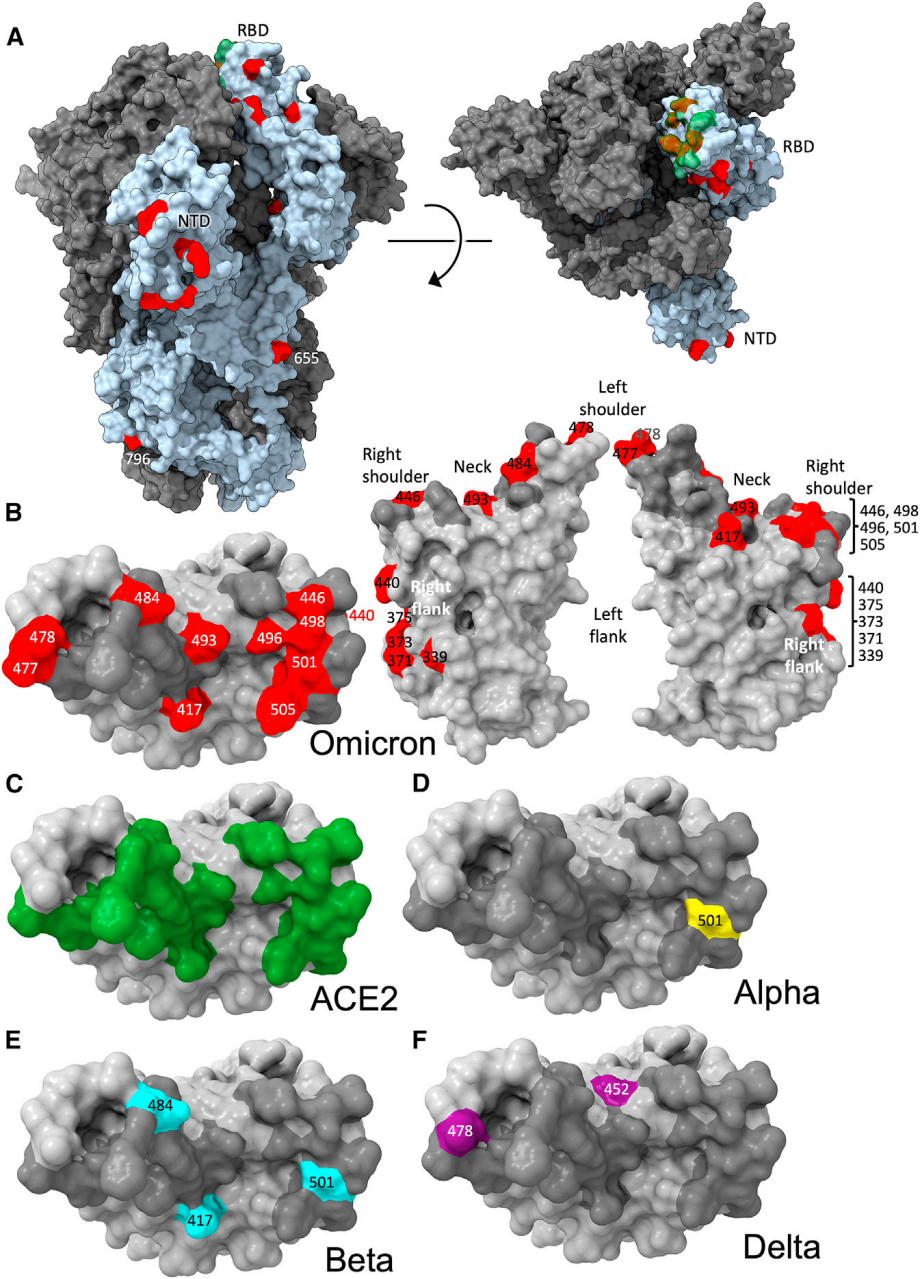
Distribution of Omicron changes (A) Trimeric S model depicted as a gray surface with one monomer highlighted in pale blue, ACE2 binding site in green and changes in Omicron shown in red, left side view, right top view. (B) RBD depicted as a gray surface with the ACE2 footprint in dark gray and changes in Omicron in red, left: top view, right: front and back views. Epitopes are labeled according to the torso analogy and mutations labeled. (C–F) Top view of RBD depicted as a gray surface with the following: (C) ACE2 binding site in green. (D) Alpha change in yellow, (E) Beta changes in cyan, and (F) Delta changes in purple. Figure produced using chimeraX (Pettersen et al., 2021). Related to Figure S1.

**Figure S1:**
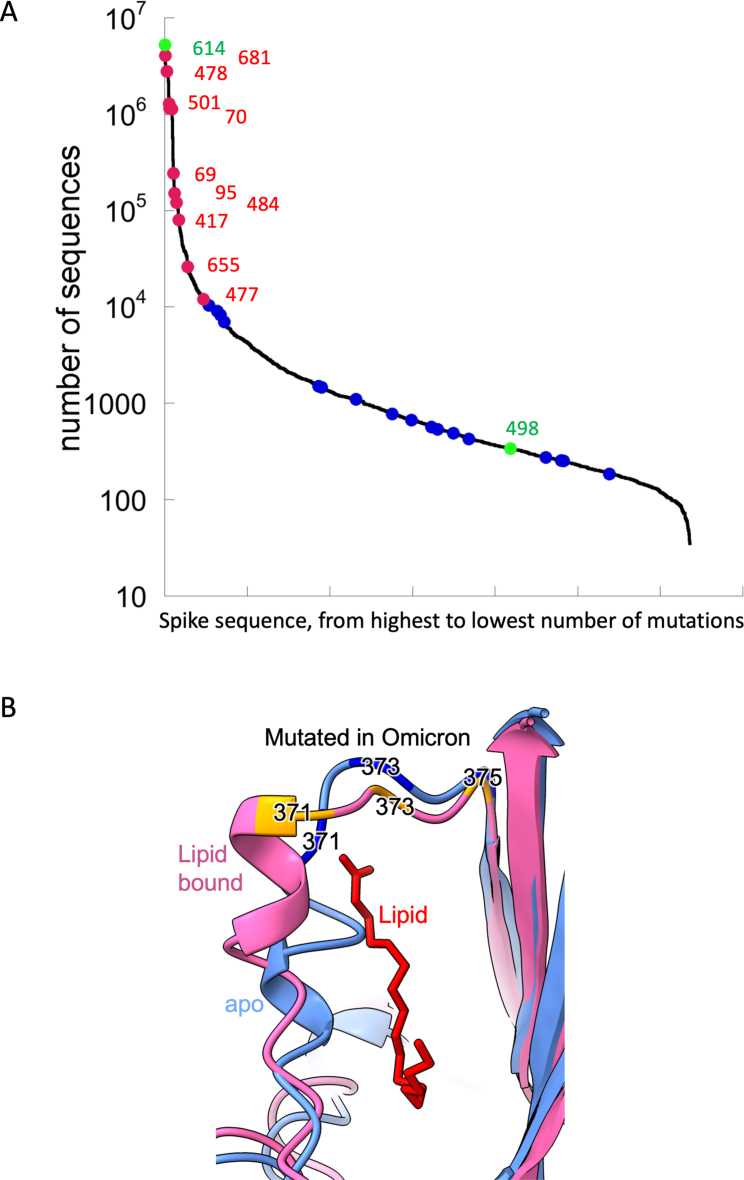
Omicron mutations, related to Figures 2 and 7E (A) Number of sequenced mutations per position. The line shows the number of mutations per residue, for high to low along the spike protein. In green are mutations D614G, which is fixed from early virus evolution and position 498, which became dominant only in Omicron. Red are mutations in Omicron identified earlier in multiple linages and blue are mutations with Omicron being the only lineage. (B) Location of the S371L, S373P, and S375F mutations in the context of the conformation change occurring on binding lipid. Cartoons of the apo (blue) and lipid bound (pink) early pandemic RBD are shown. The lipid is shown in red.

Omicron has changes throughout its proteome, but S changes dominate, with 30 amino acid substitutions plus 6 residues deleted and 3 inserted (Figures 1 and 2). Ten of these were found previously in at least two lineages (D614G was mutated early on and maintained throughout). Of those ten, six have the same amino acid substitution in >75% of the sequences, and only one (E484A) has a unique substitution in Omicron (in Beta and Gamma it is a Lys). Figure S1A shows the number of mutant sequences per residue at positions undergoing mutations in independent lineages. This can be interpreted in two ways: one is that the later mutations are epistatic to one another and thus are more difficult to reach, or that they do not contribute to virus fitness.

The Omicron RBD has 15 changes in total, as described in the next section. The NTD also has numerous changes, including 4 amino acid substitutions, 6 amino acids deleted, and 3 amino acids inserted, also described in the next section. Several mutations found in Omicron occur in residues conserved in SARS-CoV-1 and in many other Sarbecoviruses (Figure 1). These observations agree with the Pango classification (Rambaut et al., 2020), which places Omicron at a substantial distance from all other variants.

### Mapping of Omicron RBD mutations compared with Alpha, Beta, Gamma, and Delta

The Alpha variant has a single change in the RBD at N501Y (Figure 2D; Supasa et al., 2021), which occupies the right shoulder and contributes to the ACE2 binding footprint. Beta has two further mutations in the RBD: K417N and E484K, at the back of the neck and left shoulder, respectively (Figure 2E), which are also part of the ACE2 footprint (Figure 2C; Zhou et al., 2021). Gamma mutations are similar: K417T, E484K, and N501Y (Dejnirattisai et al., 2021b). Delta mutations: L452R in front of the neck, and T478K on the far side of the left shoulder, fall just peripheral to the ACE2 binding footprint (Figure 2F; Liu et al., 2021a). All of these variants have at least one RBD mutation in common with Omicron. Of the 15 Omicron changes in the RBD, nine map to the ACE2 binding footprint: K417N, G446S, S477N, E484A, Q493R, G496S, Q498R, N501Y, Y505H, with N440K and T478K just peripheral (Figures 2B and 2C). Additionally, mutations occur on the right flank: G339D, S371L, S373P, and S375F (Figure 2B), the last three of which are adjacent to a lipid-binding pocket (Figure S1B) (Toelzer et al., 2020; Carrique et al., 2020). This pocket has been seen occupied by a lipid similar to linoleic acid in an unusually rigid state of S, where all RBDs are found in a locked-down configuration stabilized by lipid-bridged quaternary interactions between adjacent RBDs. However, this lipid-bound form has been seen rarely; instead, the pocket is usually empty and collapsed, with the RBD alternating between looser down and up conformations. We presume that this is because the pocket readily empties of lipid during protein purification. Indeed, rapidly prepared virus particles tend to have RBDs closer to locked-down state (Ke et al., 2020). Loss of lipid promotes RBD presentation to the target cell.

Until now, the antigenic properties of variant viruses have been well described by assuming that each mutation produces only a local change in structure, and we used this assumption to rationalize the serological impact of the mutations in Omicron. We present structural data later to qualify this assumption.

### Omicron NTD mutations

The mutations seen in the NTD lie on exposed flexible loops, which differ from those in SARS-CoV-1 and are likely antigenic (Figure 1A). The pattern of deletions and insertions seen in Omicron consistently changes those loops that are most different from SARS-CoV-1 to be more SARS-CoV-1-like, at least in length. Of the N1, N3, and N5 loops, which comprise the antibody supersite, Omicron has a substitution at G142D and deletion of residues 143–145 in N3, which would mitigate against binding by a number of potent neutralizing antibodies, e.g., 4A8 and mAb 159 (Chi et al., 2020; Dejnirattisai et al., 2021b). The deletion of residues 69 and 70 in N2 has also occurred in the Alpha variant, whereas the deletion at residue 211, the substitution at 212, and the insertion at 214 are unique to Omicron. All these changes are on the outer surface and are likely antigenic.

### Neutralization of Omicron by convalescent serum

We isolated Omicron virus from the throat swab of an infected case in the UK. Following culture in VeroE6 cells transfected with TMPRSS2, the S gene sequence was confirmed to be the Omicron consensus with the additional mutation A701V, which is present in a small number of Omicron sequences.

We have collected convalescent serum/plasma with the indicated median day of sampling, from individuals infected early in the pandemic (n = 32, median day 42) before the emergence of the VOC, along with cases infected with Alpha (n = 18, median day 18), Beta (n = 14, median day 61), Gamma (n = 16, median day 63), and Delta (n = 42, median day 38). Neutralization assays were performed against Omicron and compared with neutralization titers for Victoria (an early pandemic strain), Alpha, Beta, Gamma, and Delta.

**Figure 3:**
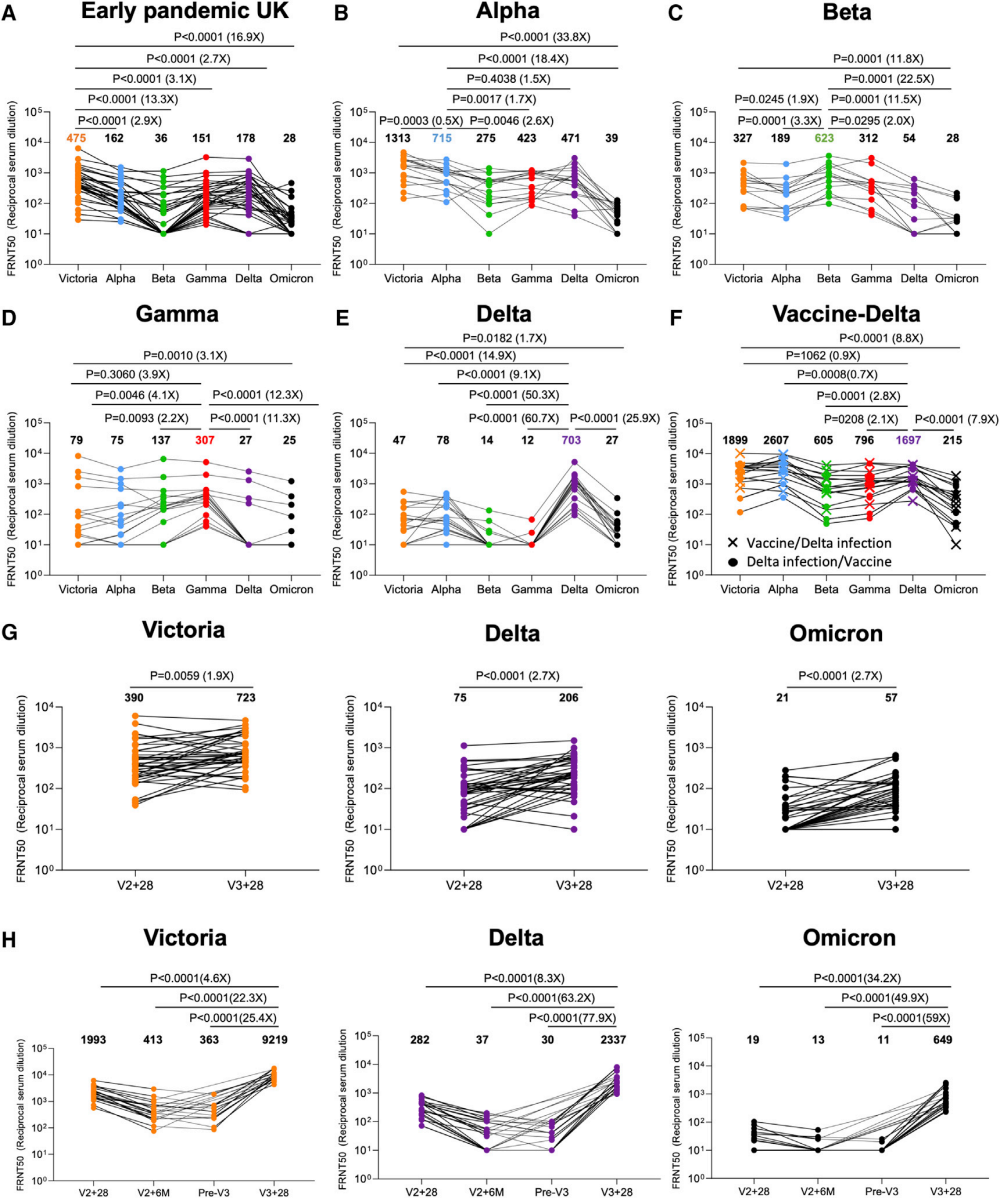
Neutralization assays against Omicron (A–H) FRNT50 values for the indicated viruses using serum from convalescent subjects previously infected with (A) early pandemic virus (n = 32), (B) Alpha (n = 18), (C) Beta (n = 14), (D) Gamma (n = 16), (E) Delta (n = 19), (F) Delta before vaccination or Delta after vaccination (n = 17), (G) before and after the third dose of AZD1222 (n = 41), and (H) 4 weeks, 6 months after the second dose, before the third, and after the third dose of BNT162b2 (n = 20). In (A–E) comparison is made with neutralization titers to Victoria, Alpha, Beta, Gamma, and Delta previously reported in Dejnirattisai et al. (2021a, 2021b), Supasa et al. (2021), Zhou et al. (2021), and Liu et al. (2021b), in (G) the data points for Victoria and Delta titers on BNT162b2 are taken from Flaxman et al. (2021). Geometric mean titers are shown above each column. The Wilcoxon matched-pairs signed rank test was used for the analysis, and two-tailed p values were calculated.

In all cases, neutralization titers to Omicron were substantially reduced compared with either the ancestral strain Victoria or the homologous strain causing infection, and in a number of cases, the immune serum failed to neutralize Omicron at 1/20 dilution (Figures 3A–3E). Compared with Victoria, the neutralization titers of sera for Omicron were reduced for early pandemic 16.9-fold (p < 0.0001), Alpha 33.8-fold (p < 0.0001), Beta 11.8-fold (p = 0.0001), Gamma 3.1-fold (p = 0.001), and Delta 1.7-fold (p = 0.0182). Compared with the neutralization of homologous viruses, for example, Alpha virus by Alpha serum, Omicron neutralization was reduced for sera from Alpha 18.4-fold (p < 0.0001), Beta 22.5-fold (p < 0.0001), Gamma 12.3-fold (p < 0.0001), and Delta 25.9-fold (p < 0.0001).

In summary, Omicron causes widespread escape from neutralization by serum obtained following infection by a range of SARS-CoV-2 variants, meaning that previously infected individuals will have little protection from infection with Omicron, although it is hoped that they will still maintain protection from severe disease.

### Vaccination and infection in combination increases Omicron neutralization titers

**Figure S2:**
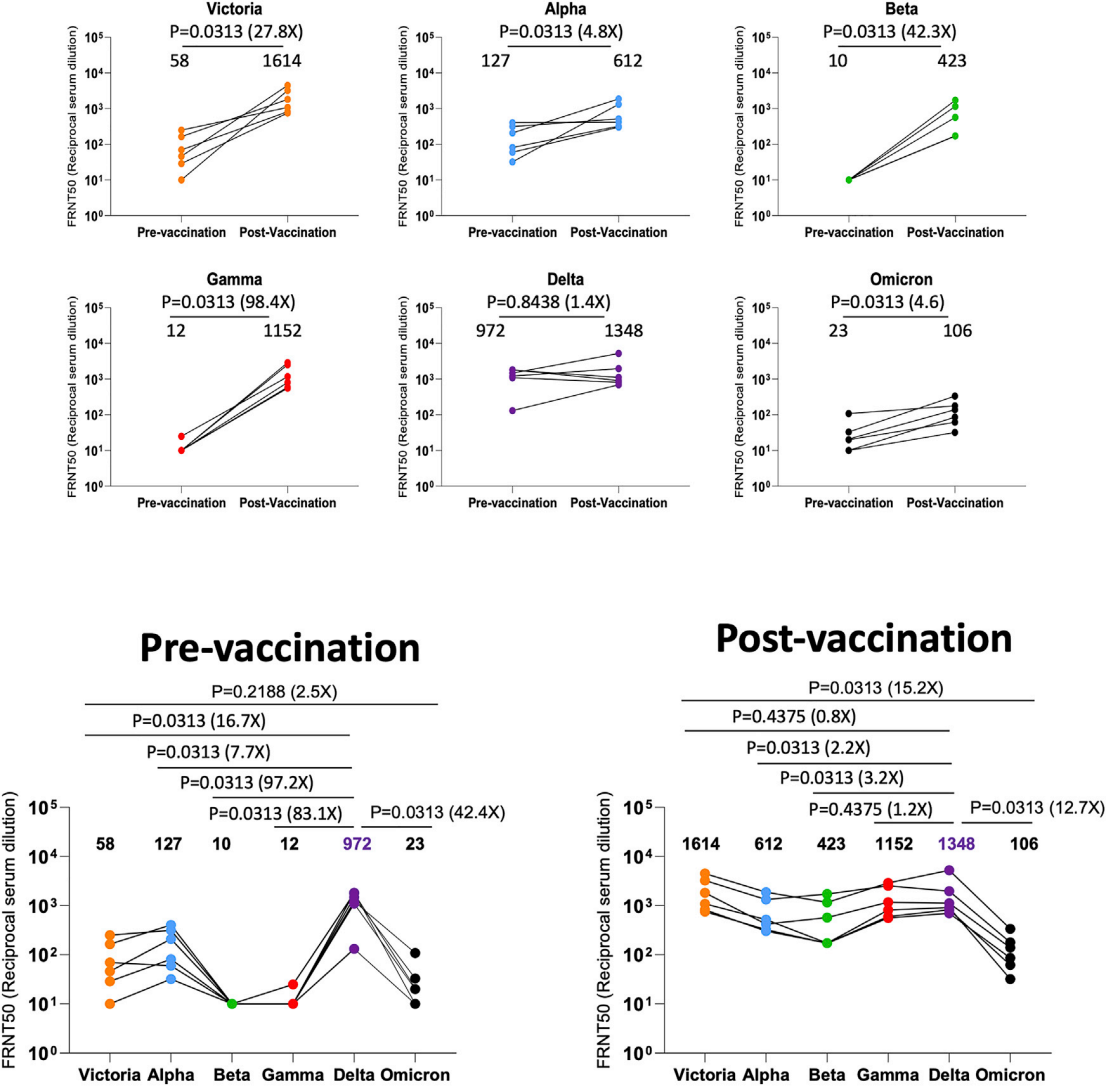
FRNT50 values for 7 cases of Delta infection before and after vaccination, related to Figure 4

We have collected sera from Delta-infected cases and because Delta spread in the UK during the vaccination campaign, we obtained sera from three different groups—Delta infection only (n = 19) (Figure 3E), Delta infection following vaccination (n = 9), and vaccination following Delta infection (n = 8) (Figure 3F). Neutralization assays against early pandemic, Alpha, Beta, Gamma, Delta, and Omicron viruses were performed. Compared with Delta-only infected individuals, sera from cases who had received the vaccine and been infected by Delta showed substantially higher neutralization to all viruses tested—early pandemic, with Delta-infected and vaccinated sera showing a 7.9-fold (p < 0.0001) increase in the neutralization of Omicron compared with Delta infection alone. To confirm the boosting effect of vaccination, we collected a paired blood sample from 6 Delta cases before and after vaccination, which clearly demonstrated the boosting effect of infection and vaccination (Figure S2).

### Increased neutralization of Omicron by third-dose booster vaccination

In a number of countries, booster programs have been launched to counter waning immunity and the increasing frequency of breakthrough infections with Delta. To examine the effect of booster vaccination, we tested neutralization of Victoria, Delta, and Omicron viruses using sera from individuals receiving 3 doses of ADZ1222 (n = 41) or BNT162b2 (n = 20). For ADZ1222, the serum was obtained 28 days following the second and third doses (Figure 3G). For BNT162b2, the serum was obtained 28 days, 6 months, immediately prior to the third dose, and 28 days following the third dose (Figure 3H).

At 28 days following the third dose, for ADZ1222, the neutralization titer to Omicron was reduced 12.7-fold (p < 0.0001) compared with Victoria and 3.6-fold (p < 0.0001) compared with Delta; for BNT162b2, the neutralization titer to Omicron was reduced 14.2-fold (p < 0.0001) compared with Victoria and 3.6-fold (p < 0.0001) compared with Delta. The neutralization titers for Omicron were boosted 2.7-fold (p < 0.0001) and 34.2-fold (p < 0.0001) following the third dose of ADZ1222 and BNT162b2, respectively, compared with 28 days following the second dose. Of concern, and as has been noted previously, neutralization titers fell substantially between 28 days and 6 months following the second dose of the BNT162b2 vaccine, although we did not measure titers at 6 months following the second dose of AZD1222.

In summary, neutralization titers against Omicron are boosted following a third vaccine dose, meaning that the campaign to deploy booster vaccines should add considerable protection against Omicron infection.

### Effect of Omicron mutations on antibodies elicited by early pandemic virus

**Figure 4:**
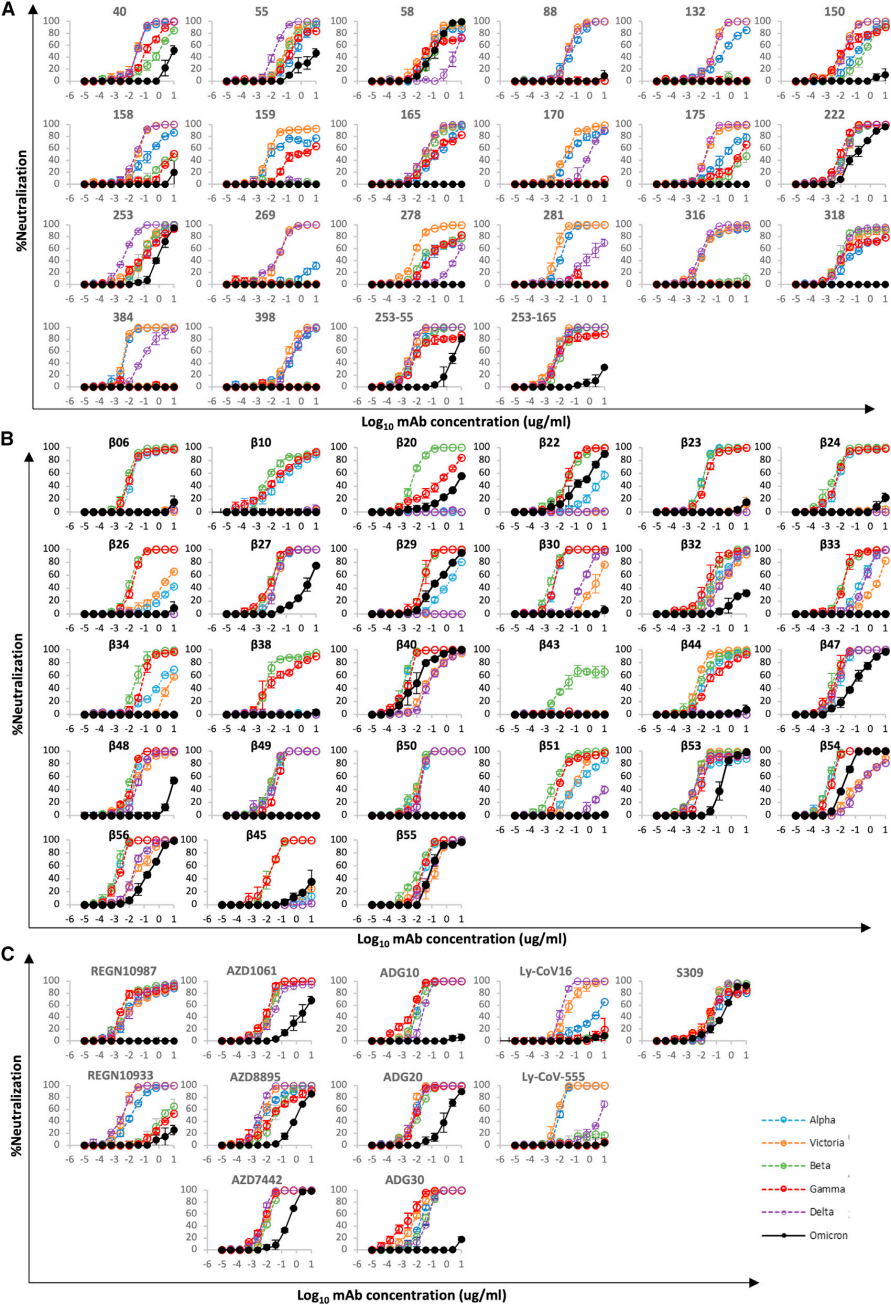
mAb neutralization curves (A–C) FRNT curves for mAb from (A) early pandemic, (B) Beta infected cases or (C) commercial sources. Omicron neutralization is compared with curves for Victoria, Alpha, Beta, Gamma, and Delta, which have been previously reported (Dejnirattisai et al., 2021a, 2021b; Supasa et al., 2021; Zhou et al., 2021; Liu et al., 2021b). Neutralization titers are reported in Table S1. Related to Figure S2.

We have previously reported a panel of 20 potent neutralizing antibodies (50% focus reduction in neutralization test [FRNT50] < 100 ng/mL) isolated from cases infected with early pandemic viruses (Wuhan) (Dejnirattisai et al., 2021a). Neutralization assays against Omicron were performed and compared with neutralization assays of early pandemic, Alpha, Beta, Gamma, and Delta viruses—17/20 mAbs failed to neutralize Omicron (FRNT50 > 10 μg/mL), while the titers for mAbs 58, 222, and 253 were reduced 3.4-, 12.6-, and 19.3-fold, respectively, compared with Victoria (Figure 4; Table S1).

**Figure 5:**
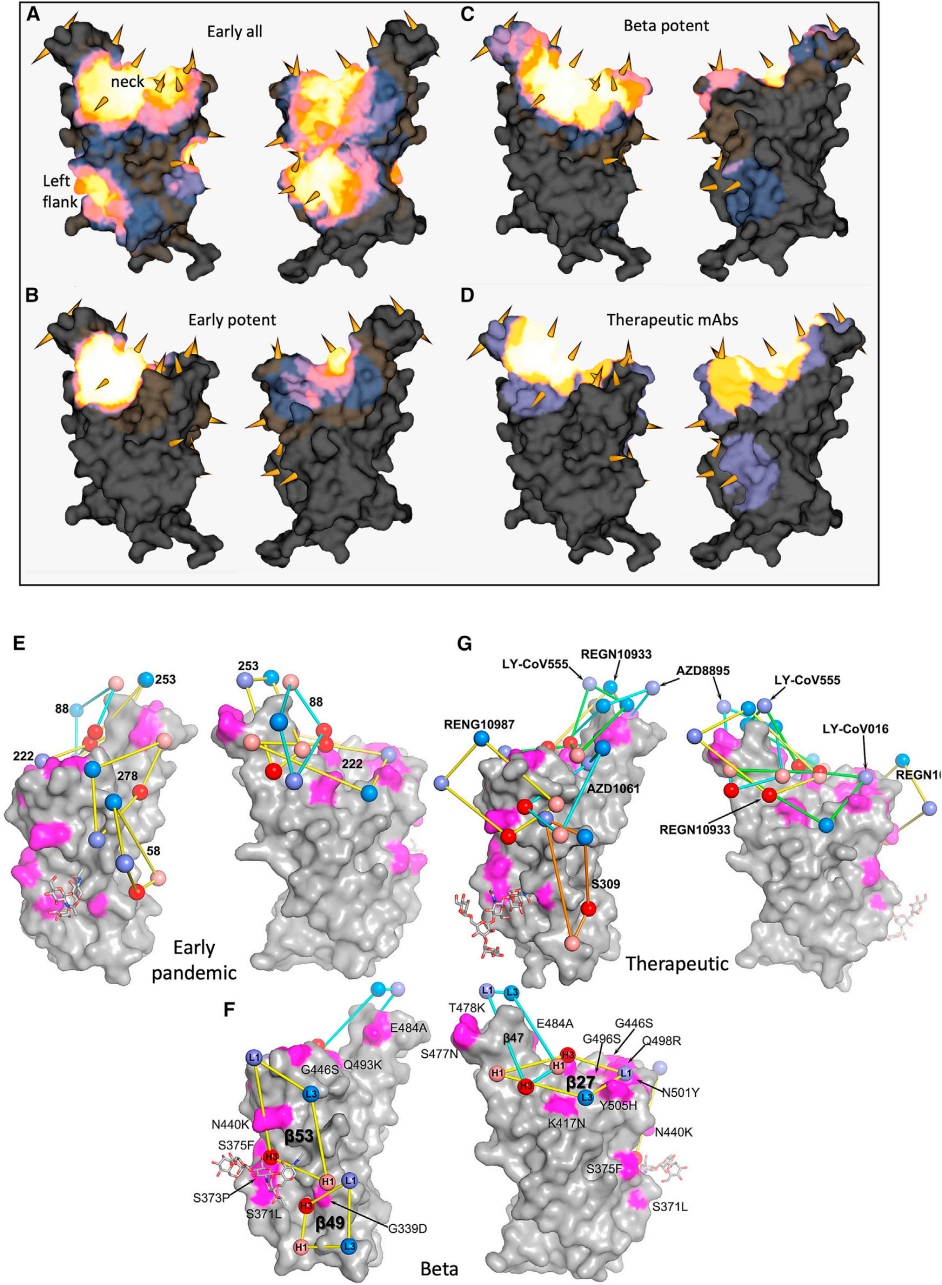
Relative antibody contact (A–D) RBD surface produced in PyMOL and rendered in mabscape using iron heat colors (gray < blue < glowing red < yellow < white) to indicate relative levels of antibody contact. Antibody contact is calculated for each surface vertex as the number of antibodies within a 10 Å radius by their known or predicted positions from earlier mapping studies (Dejnirattisai et al., 2021a; Liu et al., 2021b). Outward facing cones are placed at the nearest vertex to each mutated residue on the RBD surface. Drawn back and front views for (A) all RBD-reactive antibodies isolated from early pandemic, (B)strongly neutralizing antibodies (<100 ng/mL) from early pandemic. (C) strongly neutralizing antibodies isolated from Beta infected cases and (D) therapeutic antibodies for clinical use (from PDB: 7BEP, 6XDG, 7L7E, 7KMG, 7KMH). (E–G) Front (right) and back (left) views of the RBD drawn as a gray surface with Omicron changes highlighted in magenta and glycans drawn as sticks. (E–G) (E) Outline footprints of a selection of early pandemic mAbs: 58, 88, 222, 253, and 278 are shown by balls representing the centroid of H3 (red), H1(salmon). L3 (blue) and L1 (slate) loops joined by yellow or cyan sticks. (F) As for (E), showing a selection of Beta antibodies: 27, 47, 49, and 53. (G) As for (E) showing a selection of commercial antibodies: REGN10933, REGN10987, S309, AZD1061, AZD8895, LY-CoV555, and LY-CoV016. Related to Figures S3, S4, and S5.

The binding sites of these antibodies were mapped together with other published structures to 5 epitopes (based on the position of the center of gravity of each antibody) either by direct structural studies or competition analyses (Dejnirattisai et al., 2021a). According to the torso analogy (Dejnirattisai et al., 2021a), these were designated as follows: neck, left shoulder, right shoulder, right flank, and left flank (Figure 2B). In Figures 5A–5D, we show the mapping of the density of centroids to the surface of the RBD with the Omicron mutations shown as spikes (the information is also mapped to the primary structure in Figure 1A), and selected antibody binding is shown schematically in Figures 5E–5G. As expected, there is a correlation between the positions of the mutations and the sites of antibody binding, although the antibody centroids are more broadly spread across the RBD surface. In particular, there are no mutations in the left flank epitope, where a significant number of antibodies bind (Figure 5A). These antibodies can neutralize in some assays and confer protection (Barnes et al., 2020; Dejnirattisai et al., 2021a; Huang et al., 2021); therefore, this cryptic epitope might be an important target for therapeutic antibody applications and cross-protective vaccine antigen (Pinto et al., 2020). We demonstrate the continued binding of this class of antibodies later on.

**Figure S3:**
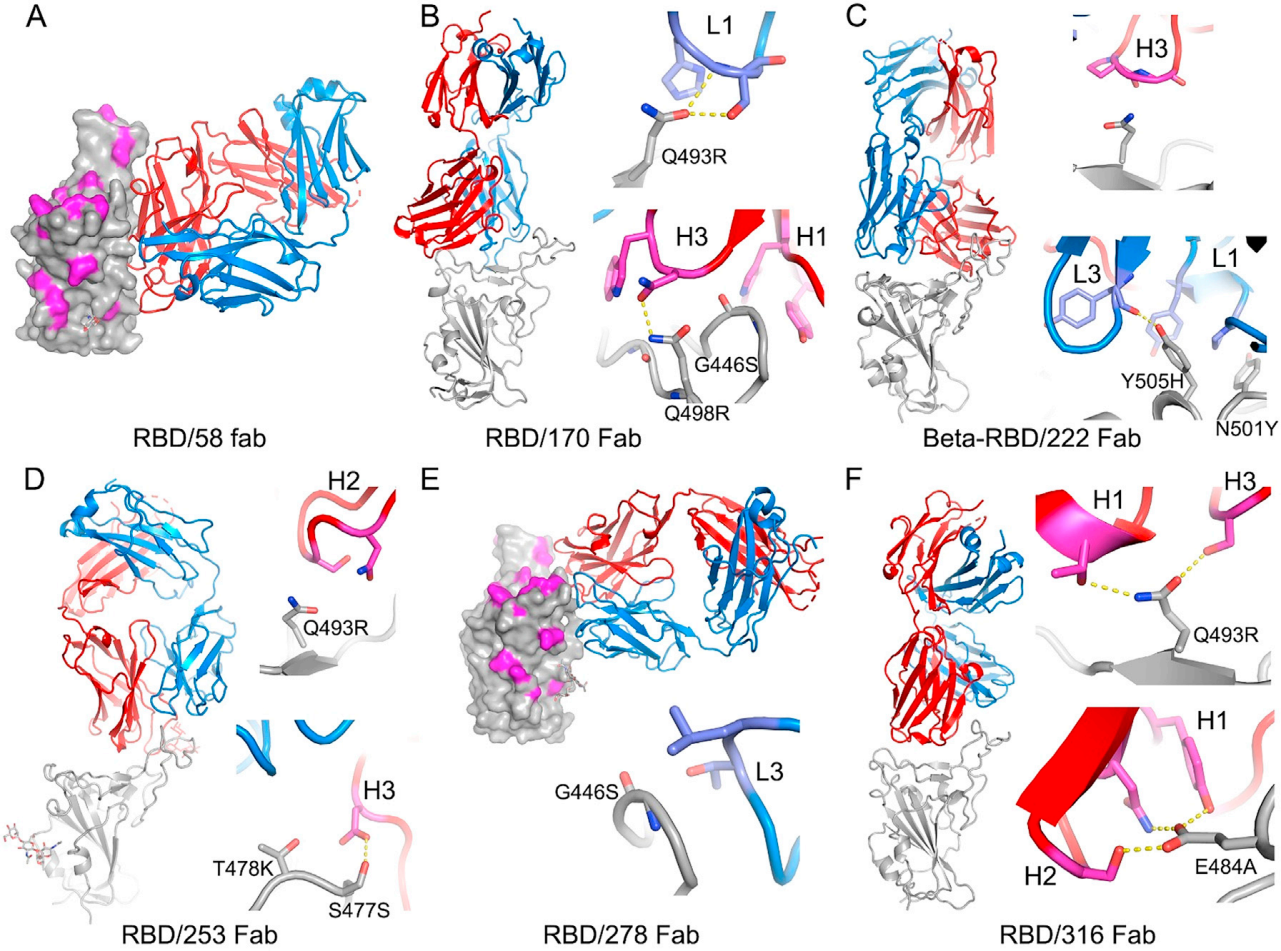
Binding modes of early pandemic mAbs and their contacts to Omicron mutation sites, related to Figure 5 (A) Fabs are drawn as ribbons with the heavy chains in red and light chains in blue and RBDs as gray ribbon or surface representation with Omicron mutation sites highlighted in magenta. Side chains are shown as sticks and hydrogen bonds as dashed lines. (A) Fab 58 does not make any close contacts with the Omicron mutation sites. (B–F) Binding modes and contacts with Omicron mutation sites of Fabs 170, 222, 253, 278, and 316, respectively.

Nineteen of the twenty most potent (FRNT50 < 100 ng/mL) neutralizing mAbs are mapped to the ACE2 binding site across the neck and shoulder epitopes of the RBD, and 5 of these are classified as public IGVH3-53 (immunoglobulin heavy chain variable gene family) antibodies (Dejnirattisai et al., 2021a; Yuan et al., 2020). Mapping these onto the RBD surface (Figure 5B) shows that the centroids are highly concentrated in the neck region. IGVH3-53 mAbs were especially common in early pandemic responses, and although their centroid is at the neck, they are orientated in such a way that their light chain CDRs interact with the right shoulder (Figure S3). Most IGVH3-53 mAbs are sensitive to the N501Y mutation, although some, such as mAb 222 or Beta-27, can still neutralize 501Y-containing viruses (Dejnirattisai et al., 2021b; Liu et al., 2021b). Omicron mutation Y505H has a direct interaction with the L1 and L3 CDRs of mAb 222 (Dejnirattisai et al., 2021b) and, together with Q493R, is likely responsible for the 12.6-fold reduction in the neutralization titer of mAb 222 (Figure 4A; Table S1).

MAbs 253, 55, and 165 are IGVH1-58 mAbs, which bind an epitope toward the left shoulder. H3 contacts S477N, and Q493R is likely disruptive of H2 interactions, leading to the 19.3-fold reduction in neutralization (Figure S3).

The neutralizing activity of mAbs 88, 316, and 384 is knocked out for Omicron (Figure 4A; Table S1); all interact with E484 (mAb 316 via H1 and H2) within the left shoulder epitope, and the E484A mutation is unfavorable. For mAb 316, Q493R will also likely be deleterious due to contacts with H1 and H3. Broadly neutralizing mAb 58 binds at the front of the RBD, reaching toward the right flank in an area that is relatively clear of mutations and thus is unaffected (Figure S3). MAb 278 binds more of the right shoulder, with L3 in contact with G446, and the G446S mutation in Omicron knocks out activity (Figure S3).

MAb 170 will be affected by Q493R and Q498R, which directly interact with L1 and H3, respectively (Figure S3). Q498R is between G496S and G446S (Figure 2B), and G446 is in proximity to H1; together, these mutations knock out the activity of mAb 170 (Figure 4A; Table S1). The binding sites of selected potent antibodies are shown in Figure 5E. All of these, with the exception of mAb58, are affected by the mutations in Omicron. To understand the resilience of mAb58, we determined the structure of a ternary complex of an early pandemic RBD with Fabs for mAbs 58 and 158 (Table S2), confirming that its epitope includes no residues mutated in Omicron (Figure S3).

### Effect of Omicron mutations on antibodies elicited by the Beta variant

We derived a panel of 27 potent Beta antibodies (FRNT < 100 ng/mL) (Liu et al., 2021b), and this revealed a surprisingly skewed response with 18/27 potent antibodies targeting the Beta mutations: E484K, K417N, and N501Y. This is seen in Figure 5C, where the focus on residues in the shoulders has spread the centroid patch out toward several Omicron mutation sites. This information is mapped to the primary structure in Figure 1A, and a schematic of the binding of the four potent cross-reactive antibodies is shown in Figure 5F. While K417N and N501Y are conserved in Omicron, E484 is mutated to an alanine, which seems a likely escape mutation from either 484E (early pandemic/Alpha) or 484K (Beta).

Neutralization assays were performed against Omicron and they showed a complete loss of activity for 17/27 Beta mAbs (Figure 4B; Table S1). Substantial reductions in neutralization titers were observed for many of the rest of the Beta panel, with Beta-22, -29, -40, -47, -53, -54, -55, and -56 being able to neutralize Omicron with titers <400 ng/mL.

**Figure S4:**
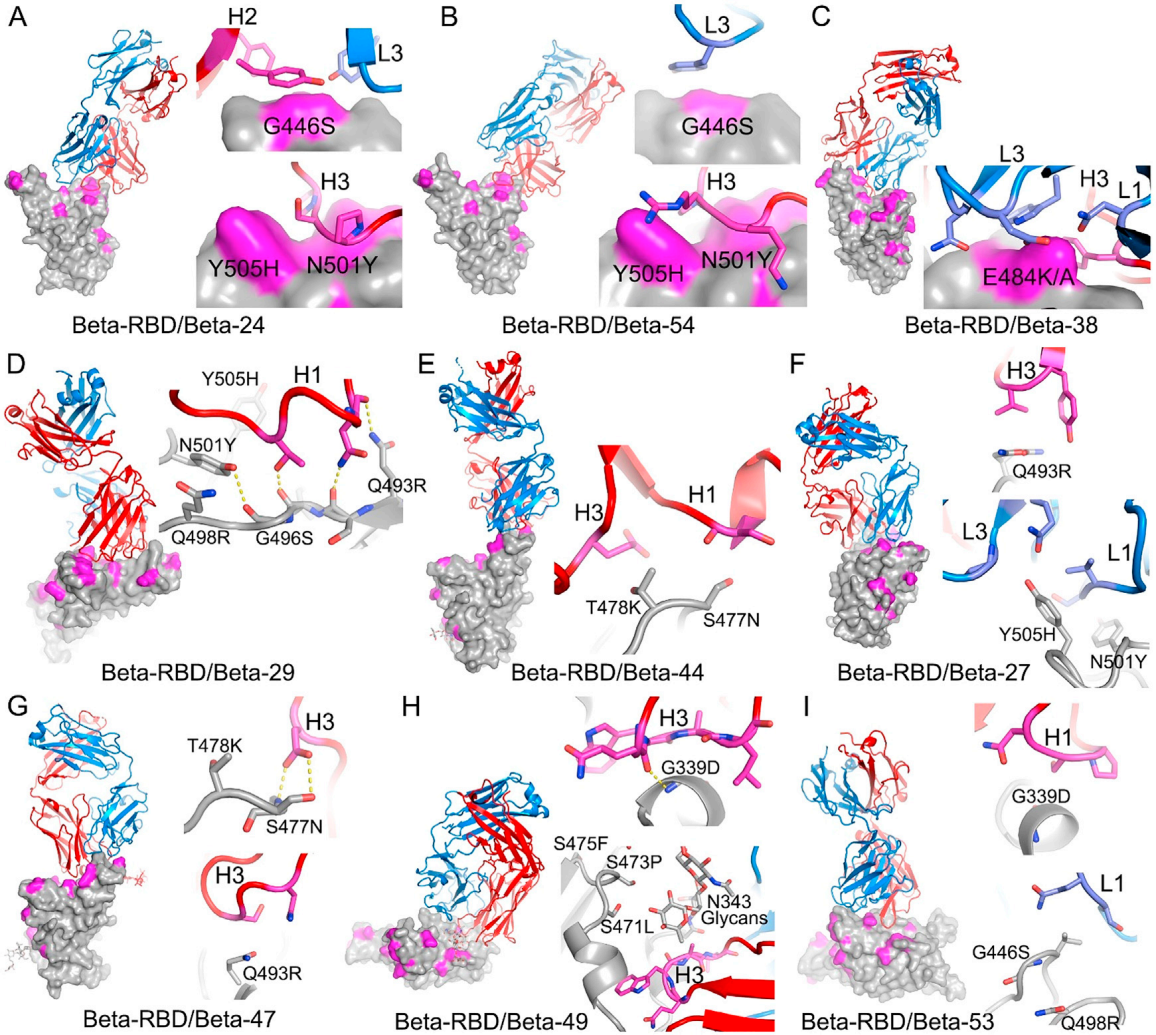
Binding modes of Beta mAbs and their contacts to Omicron mutation sites, related to Figure 5 (A and B) The drawing and coloring schemes are same as in Figure S3. These are structures of Beta-RBD/Beta-Fab complexes. (A) Beta-24 and (B) Beta-54, examples of Beta mAbs targeting the N501Y mutation site. (C) Beta-38, a representative of Beta mAbs targeting the E484K mutation site. (D) Beta-29, a K417N/T-dependent Beta mAb. (E) Beta-44 binds at the top of left shoulder and is sensitive to T478K mutation. (F–I) Beta-27, -47, -49, and -53, respectively. These four Beta mAbs neutralize all the previous variants of concern, as well as the early pandemic Wuhan strain.

A large number of Beta mAbs target the 501Y mutation, including a public antibody response mediated through IGVH4-39 (n = 6) and the related IGVH4-30 (n = 1) (Liu et al., 2021b). Many are likely to be sensitive to the numerous mutations in this region: N440K, G446S, Q493R, G496S, Q498R, and Y505H. In total, 11 antibodies contact 501Y; *Beta**-6**, -**10**, -**23**, -**24**,* -**30***, -40, -54, -55,* and -56, while Beta-22 and -29 bind epitopes dependent on 417N/T together with 501Y (antibodies in italic are VH4-39 or VH4-30 and the neutralization of Omicron for those in bold is completely knocked out). Beta mAbs targeting the back of the neck epitope (Beta-22, -29, and -30) will be affected, for example, in the case of Beta-29, H1 makes extensive interactions with residues Q493, G496, and Y505 (Figure S4). Beta-44 binding to the left shoulder epitope has already been shown to be sensitive to T478K, while the combination of S477N and T478K in Omicron is likely to be more deleterious. Interestingly, several of the antibodies (Beta-40*,* -54, -55, -56, and Beta-22 and -29 [501Y 417N/T]) retain some activity, and this is explained later on with reference also to the structure of the Omicron RBD/Fab 55 complex.

Four Beta mAbs potently cross-neutralize all Alpha, Beta, Gamma, and Delta variants (Liu et al., 2021b, Figure 5F). Of these, Beta-27 is a VH3-53 antibody that contacts Q493 and Y505 in a similar way to mAb222 and shows reduced neutralization of Omicron (Figure 4B; Table S1). Beta-47, a VH1-58 antibody, has contacts with S477 and Q493, likely leading to the observed reduction in neutralization of Omicron.

Beta-49 and -50, which belong to the IGVH1-69 gene family, bind similarly to the right flank and are knocked out by Omicron (Figure 4B; Table S1). They lie directly on RBD G339 and would clash with G339D. Beta-53 also binds to the right flank, with H1 contacting residue 339 and likely clashing with G339D. L1 likely contacts G446S, leading to the observed two-log reduction in Omicron neutralization compared with Beta (Figure S4).

### Effect of Omicron mutations on current antibody therapeutics

**Figure S5:**
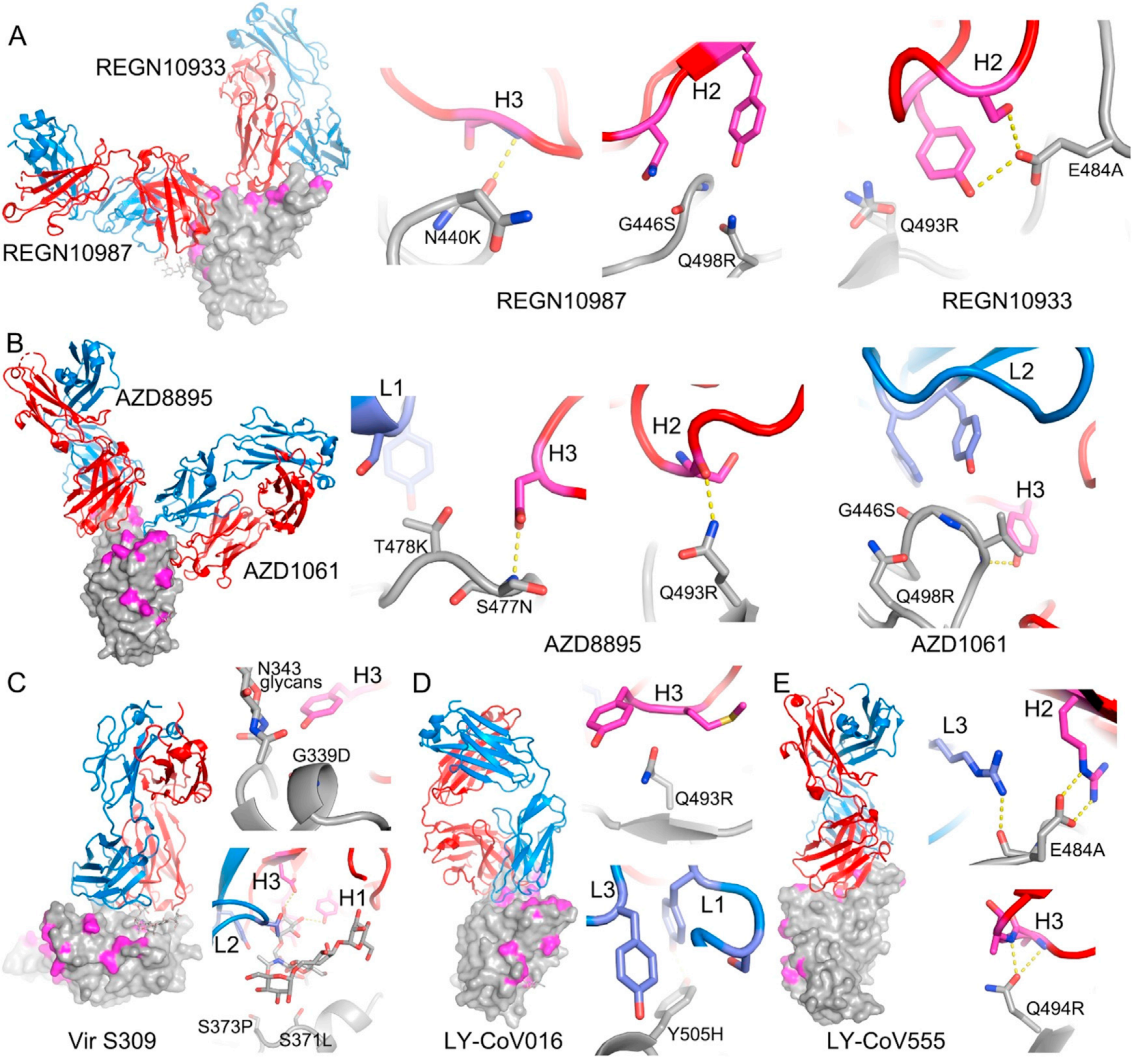
Binding modes of the therapeutic mAbs and their contacts to Omicron mutation sites, related to Figure 5 (A–E) The drawing and coloring schemes are same as in Figure S3. (A) REGN10987 and REGN10933, (B) AZD8895 and AZD1061, (C) Vir S309, (D) LY-CoV016, and (E) LY-CoV555.

Various individual antibodies or cocktails of antibodies (usually recognizing different epitopes to reduce the risk of escape [Sun et al., 2021]) have been licensed for use, and the aggregate of their binding is shown in Figure 5G. This illustrates the strong correlation of binding with sites of mutation (this is mapped to the primary structure in Figure 1A) and neutralization of Omicron is markedly reduced in most (Figure 4C; Table S1). Specifically, they are as follows:

*Regeneron 10933 and 10987*: Regeneron 10933 (Weinreich et al., 2021) binds to the back of the left shoulder and 10987 to the right shoulder ( Figures 5G and S5); activity of both is knocked out on Omicron (Figure 4C). REGN10933 is unable to effectively neutralize Beta, being sensitive to E484K (Zhou et al., 2021), and H2 contacts Q493, so that neutralization of Omicron is almost completely lost. REGN10987 contacts N440 and G446 causing complete loss of neutralization (Figure S5).

**Figure S6:**
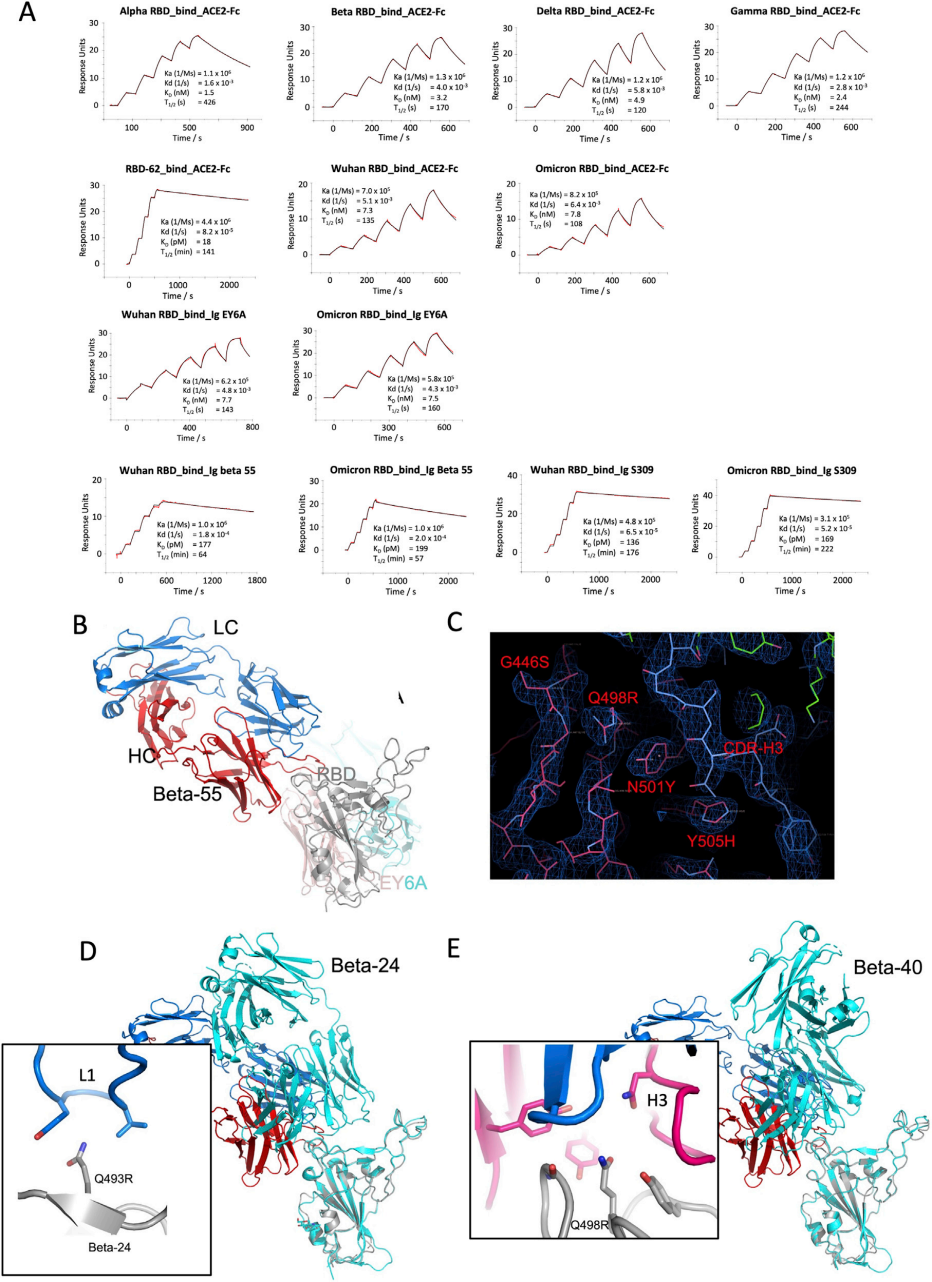
SPR measurement and crystal structure of the Omicron RBD complexed with Beta-55 and EY6A Fabs, related to Figure 7 (A) SPR measurements. (B) Ternary complex of the Omicron RBD (gray)/Beta-55 (heavy chain red, light chain blue)/EY6A (heavy chain salmon, light chain cyan). (C) Electron density map showing the density for the mutated residues at 446, 498, 501, and 505, and their interactions with the CDR-H3 of Beta-55. (D and E) Comparison of the slightly different binding mode of Beta-55 to Beta-24 (cyan in D) and Beta-40 (cyan in E), the close-up boxes show details of the interactions with Beta-24 and Beta-40 explaining the knockout of Beta-24 and the resilience of Beta-40.

*Vir S309*: S309 (Dejnirattisai et al., 2021a; Pinto et al., 2020; Sun and Ho, 2020) binds to the right flank, contacting G339 and N343 (glycan close to SLS371L, S373P, and S375F) (Figures 5G and S5). S309 neutralization of Omicron is only reduced 6.4-fold compared with Victoria, and binding, measured by surface plasmon resonance (SPR) measurements, is little affected (Figure S6A).

*AZD8895 and AZD1061*: AZD8895, a VH1-58 antibody, binds to the back of the left shoulder and activity on Omicron is reduced 230-fold compared with Victoria due to contacts with S477 (H3) and Q493 (H2). AZD1061, binding the front of the right shoulder is reduced 268-fold (Figures 5G and S5) due to L2 and H3 contacts with the G446 loop. AZD7442 (a combination of AZD8895 and AZD1061) maintains neutralizing activity against Omicron, although reduced 30.3-fold compared with Victoria.

*LY-CoV016 and 555*: The activity of both antibodies on Omicron is knocked out. LY-CoV016 is a VH3-53 antibody and extensive interactions with N501 and Y505 via L1 and L3 make it vulnerable to mutations at these residues (Figures 5G and S5). LY-CoV555 (Sun and Ho, 2020) is sensitive to the E484K mutation in Delta (Liu et al., 2021a) and also contacts Q493.

*ADG 10, 20, 30*: All Adagio antibodies suffer considerable loss of activity against Omicron (Figure 4C). The activity of ADG10 and ADG30 were completely lost, while ADG20 activity was reduced 276-fold.

### Effect on RBD/ACE2 interaction

**Figure 6:**
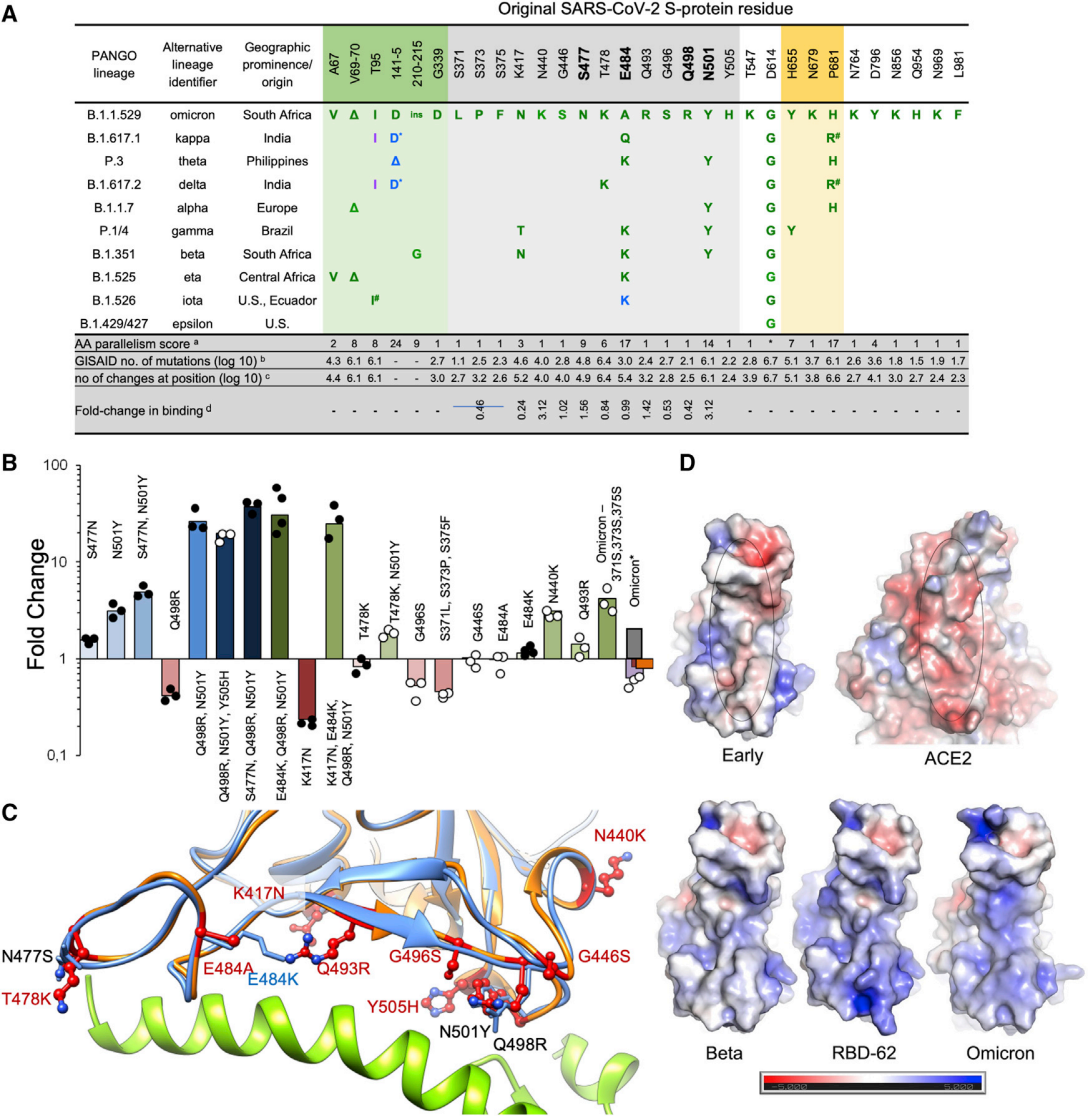
Affinity driving mutations in Omicron RBD have previously been identified by *in vitro* evolution for tighter binding (A) Analysis of the occurrence and prevalence of Omicron-variant mutations. The background is colored according to S-protein functional domains. The four positions critical for the high affinity of RBD-62 are highlighted in bold. Mutation frequencies within individual lineages are denoted in green (100%–75%), blue (75%–50%), and magenta (50%–25%). Information about the distribution and frequency of S-protein mutations and the spatiotemporal characterization of SARS-CoV-2 lineages were retrieved from www.outbreak.info (Mullen et al., 2020) and GISAID database (Elbe and Buckland-Merrett, 2017).^∗^ Same evolutionary origin, ^a^number of evolutionary non-related lineages with given or similar mutation (Zahradnik et al., 2021c), ^b^log(10) number of the observed Omicron mutation at the given position as determined on 14^th^ November 2021, ^c^same as ^b^but total log(10) number of changes at the given position. ^d^Fold-change in binding as determined by yeast-surface display. Fold-change is the ratio between original RBD KD and the mutant RBD KD for binding human ACE2. (B) Comparison of fold-change in binding affinity among selected mutations and their combinations as determined by titrating ACE2 on yeast surface displayed RBD mutations. For Omicron, yeast titration is denoted in violet, SPR (this study) is dark red, SPR as determined in Cameroni et al. (2021) is gray and ELISA as determined in Schubert et al. (2021) is in orange. Data denoted by black dots have been reported previously (Zahradník et al., 2021b). (C) RBD-62 (blue)/ACE2 (green) structure (PDB: 7BH9) overlaid on Omicron RBD structure (orange) as determined bound to Beta-55. All Omicron mutations are shown, overlaid on relevant RBD-62 mutations. (D) Electrostatic potential surface depictions calculated using PyMol. Blue is positive and red negative potential (scale bar shown below).

Fitness of a virus can stem from higher infectivity or evasion of the immune system. One way to identify mutations that increase binding affinity is by selection, using a randomly mutated RBD displayed on the yeast surface for ACE2 binding to obtain the highest affinity clone RBD-62. Mutations fixed for higher affinity binding included N501Y, E484K, S477N, and most prominently, Q498R (Figure 6A; Zahradník et al., 2021b). Interestingly, Q498R was selected only at later stages. This is explained by the 2-fold reduction in affinity as a single mutation (Figure 6A). However, in combination with the N501Y mutation, the affinity is increased 26-fold, more than any other mutation analyzed. Adding to this, the S477N mutation, one obtains a 37-fold increase in binding (Figure 6B). These three mutations, selected through *in vitro* evolution, were found together for the first time in the Omicron variant.

We measured the affinity of Omicron RBD for ACE2 using SPR and yeast display titration. Perhaps surprisingly, the affinity was on par with that of the early virus, 8 and 7 nM, respectively, using SPR (Figures 6B and S6A) and 2.9 and 1.9 nM using yeast display titration (SPR and yeast display titration data strongly correlate but with a constant shift in absolute values [Zahradník et al., 2021b]). This implies that the increased affinity imparted by S477N, Q498R, and N501Y is being offset by other mutations in the ACE2 footprint. We measured the affinities of the other single mutations in the ACE2 binding footprint of Omicron (using yeast display titration), as shown in Figures 6B and 6C, and they provide a rationale for this. T478K in the presence of N501Y decreased the positive effect of the latter by 2-fold. Y505H reduces the binding of Q498R, N501Y double mutant by 50%. G496S and the triple-mutation S371L, S373P, and S375F reduce binding by 2- and 2.2-fold, respectively. The effect of changing the triple-mutant (S371L, S373P, and S375F) back to the wild-type sequence was even more pronounced in the background of Omicron, in which the affinity increased from 2.9 to 0.4 nM (7-fold). Moreover, this back-to-wild-type triple-mutant increased the expression on the surface of yeast 10-fold relative to Omicron. This indicates a functional role in increasing the fitness of the virus for this triple-mutant, which requires the binding enhancement provided by the Q498R, N501Y double mutant. E484A (instead of the Lys found in other variants, Figure 6A) was neutral. While K417N (found in the Beta variant) on its own decreases binding substantially, the effect on binding when combined with other mutations is smaller (Figure 6B). Two single mutations found specifically in Omicron, Q493R, and N440K did increase binding, probably due to increasing the electrostatic complementarity between ACE2 (negatively charged) and the RBD (positively charged) (Figure 6D).

Comparing the structure of the complex of the pM affinity RBD-62 with ACE2 (Zahradnik et al., 2021b; PDB:7BH9) to that of Omicron, RBD bound to Beta-55 antibody (described later on, see Table S2; Figure 6C) shows high similarity with an RMSD of 0.55 Å over 139 residues. Importantly, the locations of the binding-enhancing mutations 477N, 498R, and 501Y are conserved between the two, despite the RBD-62 being bound to ACE2, while Omicron RBD is not. This shows that these residues are pre-arranged for tight binding, implying low entropic penalty of binding.

### Antigenic cartography

**Figure 7:**
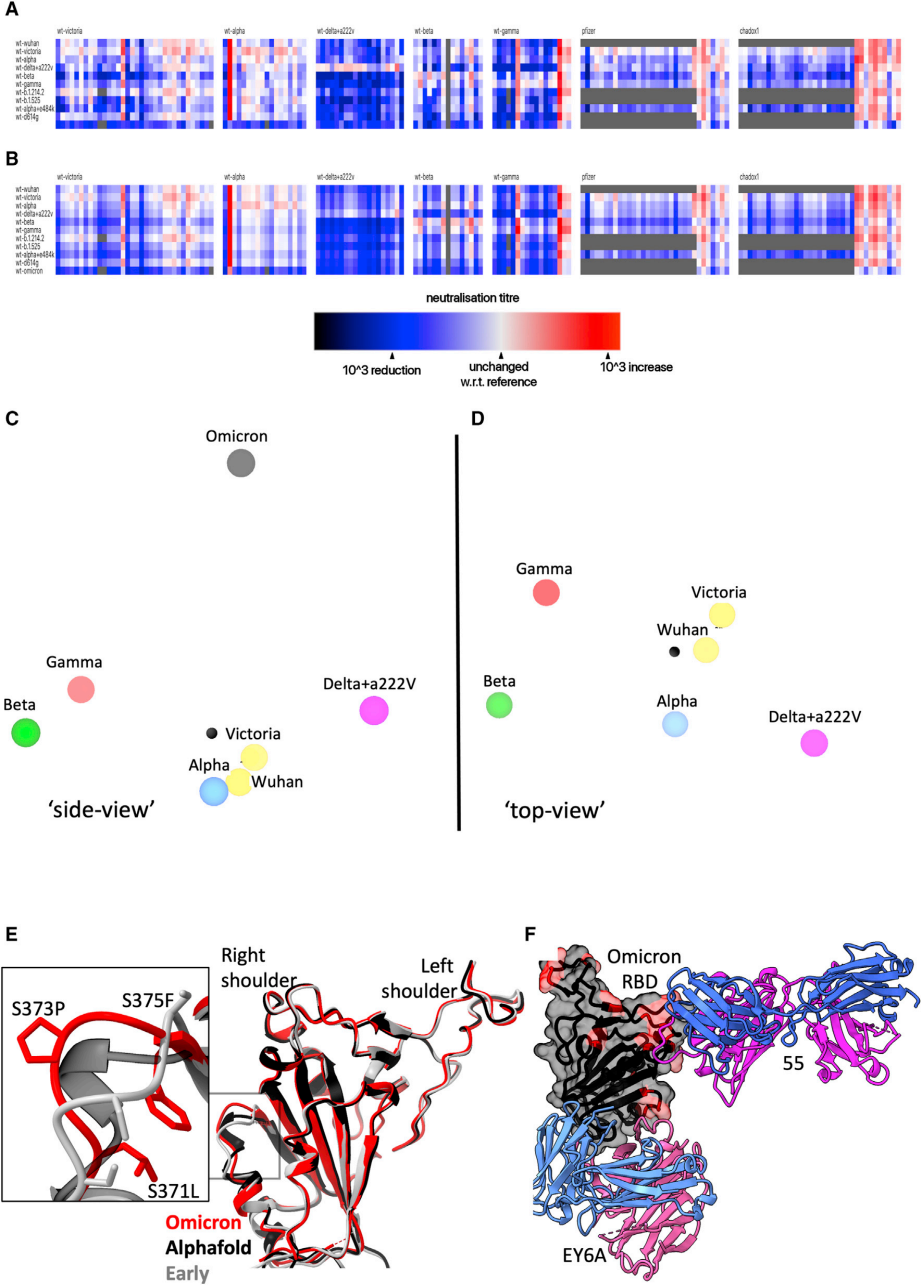
Antigenic map from neutralization data for Omicron (A) Neutralization data (log titers) showing sera as columns against challenge variants as rows. Sera are grouped into blocks according to the eliciting variant. The reference neutralization titer for each block is calculated as the average of all titers when challenged with the variant that elicited the serum. In the case of vaccine sera this was taken as the average of all best neutralization titers. Therefore, colors within a single block express the relative neutralization titer with respect to this reference. (B) Shows an example of the equivalent model generated from one run of antigenic map refinement using the same reference offsets as calculated for (A). (C) Shows a view of the three-dimensional antigenic map for variants of concern. The distance between two points corresponds to the drop-off in neutralization titer used in (B). (D) Same antigenic space as (C) but rotated 90°, to look downward form Omicron. (E) Overlay of the X-ray structure of Omicron (red) on the early pandemic (Wuhan) RBD (gray) and the predicted model of the Omicron RBD in black, drawn as cartoons. The structural change effected by the S371L, S373P, and S375F mutations is shown enlarged in the inset. (F) X-ray structure of ternary complex of Omicron RBD with Beta-55 and EY6A Fabs. The Omicron RBD is shown as a gray semi-transparent surface with mutated residues in magenta. Fabs are drawn as cartoons, heavy chain in magenta and light chain in blue. Related to Figures S1 and S6 and Video S1.

We used the matrix of neutralization data generated in Figure 3 to place Omicron on an antigenic map, with a method similar to that developed for analysis of the Delta variant (Liu et al., 2021a), where we model individual viruses independently and allow for serum-specific scaling of the responses (STAR Methods). This model works well; the measured and modeled responses are shown in Figures 7A and 7B (with 1,600 observations and 215 parameters, the residual error is 9.1%). The results are well described in three dimensions (see Video S1**)** and are shown projected into two dimensions in Figures 7C and 7D. It will be seen that the previous variants are placed in a planetary band around a central point, with Delta opposed to Beta and Gamma; however, Omicron is displaced a large distance out of this plane, almost on a line drawn from the central point perpendicular to the planetary band, illustrating vividly how Omicron dramatically expands our view of the antigenic landscape of SARS-CoV-2.

Video S1. Antigenic map for major variants of SARS-CoV-2, related to Figure 7

### The structural impact of the numerous mutations in S

We first used Alphafold2 (Jumper et al., 2021) to predict the Omicron RBD structure (Figure S1B). The top-ranked structure was very similar to the early pandemic RBD (RMSD for Cαs 0.71 Å, residues 334–528), with a significant difference in the region of the triple serine mutations 371–375, on the right flank (Figure S1B). We then determined the high-resolution crystal structure of the Omicron RBD domain in complex with two Fabs: Beta-55 and EY6A (Figure S6B and Table S2) (Huang et al., 2021; Liu et al., 2021b; Zhou et al., 2020). The RBD structure is indeed close to that of early pandemic viruses (RMSD 0.9 Å for 187 Cα) with the only significant change at the 371/373/375 triple serine mutations (Figure 7E). The rearrangement in this region is essentially an amplified version of that predicted by Alphafold2, suggesting that such algorithms have some value in predicting the effect of dense mutations as seen in Omicron RBD. The mutations S371L, S373P, and S375F are all changes from small, flexible polar serine residues to bulkier, less flexible hydrophobic residues. Interestingly, all the Omicron S mutations involve single codon changes apart from S371L, which requires two changes from TCC to CTC, indicative of underlying strong selection pressure and functional change. Although the rearrangement in Omicron is quite modest, it is exactly this region of the structure that undergoes a larger conformation change when lipid is bound into the pocket (Figure S1B). Changes in the serine-rich loop allow the attached helix to swing out, opening the pocket for lipid binding. It is possible that the increased rigidity and the entropic penalty of exposing hydrophobic residues may disfavor lipid binding to Omicron, which would alter the properties of the virus, explaining the selection of these changes.

The binding of EY6A to the left flank of the RBD is essentially unchanged from that observed previously (Zhou et al., 2020) (dissociation constant [K_D_] 7.8 and 6.8 nM for early pandemic and Omicron RBDs, respectively, by SPR) (Figures 7F and S6A). This cryptic epitope, which is highly conserved for functional reasons, is a good target for broadly neutralizing therapeutic antibodies.

Beta-55, as predicted earlier (Liu et al., 2021b), binds to the right shoulder, around residue 501. Interestingly, the epitope includes several residues mutated in Omicron from the early pandemic virus (including Q498R, N501Y, and Y505H) (Figures 7E, S6B, and S6C). It is remarkable that despite these significant changes, neutralization is relatively little affected. The neutralization result was confirmed by measurements of the binding affinity, 177 pM and 204 pM for the early pandemic and Omicron RBDs, respectively (Figure S6A). To confirm the structural basis, we also determined the crystal structure of an analogous ternary complex formed with early pandemic RBD (Table S2). As expected, the details of the interaction are essentially identical. If we extend the analysis of the 501Y targeting antibodies by comparing the structures of Beta-6, -24, -40, and -54, we find subtle explanations; thus, Beta-24 and some others are knocked out due to a clash with CDR-L1 created by the Q493R mutation (Figure S6D), whereas for antibodies Beta-40, -54, and -55, this mutation can be accommodated. In addition, the Q498R mutation may create a hydrogen bond in Beta-40 or a salt bridge in Beta-54 to CDR-H3, which may compensate for the loss of binding affinity due to changes around residue 501 (Figure S6E). Thus, the surprising resilience of several of the 501Y targeting antibodies may be because the mutated residues in this region are not “hotspots” of interaction, and mutations can sometimes be accommodated without significant impact on affinity. This may suggest that a major driver for evolution was the less 501-focused responses to early viruses.

## Discussion

The first 4 Omicron sequences were deposited on 24^th^ November 2021. Within days, distant international spread was seen, and has caused great concern due to its high transmissibility and ability to infect previously exposed or vaccinated individuals. Only 3 weeks after the virus was first detected in the UK, Omicron cases outnumbered Delta in London, with the number of daily new cases in the UK larger than that recorded during any other previous time in the pandemic. Over the next weeks, disease severity will become clearer.

The density of mutational changes (including deletions and insertions) found in Omicron S is extraordinary, being more than three times that observed in previous variants. Within S, as observed for other variants, the NTD, RBD, and the furin cleavage site region are hotspots for mutation (Zahradník et al., 2021b), and within the RBD, mutations are concentrated on the ACE2 interacting surface and the right flank.

Most potent neutralizing antibodies bind on or in proximity to the ACE2 footprint (neck and shoulder epitopes) and block interaction of S with ACE2, thereby preventing viral attachment to the host cell. There are two other classes of potent neutralizing mAbs, first antibodies binding in close proximity to the N343 glycan (right flank epitope) exemplified by Vir S309 (Pinto et al., 2020), which includes the Beta-49, -50, and -53 antibodies (Liu et al., 2021b) used in our analysis. These mAbs bind distant from the ACE2 binding site, do not block ACE2 interaction, and destabilize the S-trimer, which may be their mechanism of action. Finally, antibodies binding to the supersite on the NTD can also be potently neutralizing, although the mechanism of action of NTD antibodies remains obscure (Cerutti et al., 2021; Chi et al., 2020; Dejnirattisai et al., 2021a). Multiple mutations at all three of these sites—the receptor-binding site, proximal to N343 glycan, and NTD—are found in Omicron and lead to substantial reduction in neutralization titers for naturally immune or vaccine sera, with many showing complete failure of neutralization. This, together with the widespread failure of potent mAb to neutralize Omicron, points to a driver of immune evasion for their evolution.

The left flank epitope, which is not mutated in Omicron, is used by antibodies that do not block ACE2 binding but are protective (Barnes et al., 2020; Dejnirattisai et al., 2021a; Hastie et al., 2021; Huang et al., 2021; Zhou et al., 2020). Here, we demonstrate structurally and by affinity measurements that this epitope is conserved and unchanged in Omicron.

Following repeated rounds of selection by yeast display for high ACE2 affinity, RBD-62 (I358F, V445K, N460K, I468T, T470M, S477N, E484K, Q498R, and N501Y) emerged as the highest affinity clone with a 1,000-fold increase in affinity for ACE2 from 17 nM for Wuhan RBD to 16 pM for RBD-62. It is striking that the key contributors for the high affinity of RBD-62 are present in Omicron. Interestingly, the combination of mutations K417M, E484K, Q493R, Q498R, and N501Y also emerged after 30 passages in mouse lungs (Roy Wong et al., 2021). This mouse-adapted virus was highly virulent and caused more severe disease. The appearance of E484K, Q493H/R, Q498R, and N501Y in yeast display and mouse adaptation experiments are strong indications that the tighter binding to ACE2 also facilitates more efficient transmission.

However, in Omicron, overall affinity for ACE2 is not increased, suggesting a different strategy. Since mutations S477N, Q498R, and N501Y are likely to increase ACE2 affinity by 37-fold, we hypothesize that these changes, also found in RBD-62, serve to anchor the RBD to ACE2, leaving the rest of the receptor-binding motif more freedom to develop further mutations, including those that reduce ACE2 affinity, in a quest to evade the neutralizing antibody response. Indeed, K417N, T478K, G496S, Y505H, and the triple S371L, S373P, S375F reduce affinity to ACE2 while driving immune evasion. All this is achieved with very minimal structural changes in the isolated Omicron RBD (Figure 7E).

These observations provide a valuable lesson on the plasticity of protein-protein binding sites, maintaining nM binding affinity (Cohen-Khait and Schreiber, 2016). Thus, the extreme concentration of potent neutralizing antibodies around the 25 amino acid receptor footprint of ACE2 suggests that this would be an Achilles heel for SARS-CoV-2, with ACE2 placing constraints on its variability (this is why receptor-binding sites are often hidden [Rossmann et al., 1985]). However, in practice, the extraordinary plasticity of this site, allowing it to absorb mutational change while retaining affinity for ACE2, is a potent weapon to evade the antibody response. Such camouflage of receptor-binding sites has been observed before (see, for example, Acharya et al., 1989), but it seems that by acquiring a lock on the ACE2 receptor at one point, through 498 and associated mutations, many other less energetically favorable changes can be tolerated, fueling antigenic escape. Thus, by mutating the receptor-binding site, the virus can modulate ACE2 affinity and potentially transmissibility while evading the antibody response.

How Omicron evolved is under debate. The results presented here suggest that immune evasion is a primary driver in its evolution, sacrificing affinity-enhancing mutations to optimize immune-evading mutations. This could, for instance, occur in a single immunocompromised individual, with further evolution in rural, unmonitored populations (Clark et al., 2021). Virus evolution has been previously observed in chronically infected HIV+ individuals and other immunocompromised cases, leading to the expression of the N501Y, E484K, and K417T mutations (Cele et al., 2021; Karim et al., 2021; Kemp et al., 2021). What seems beyond doubt from the ratio of nonsynonymous to synonymous mutations (only one synonymous mutation in all of S) is that the evolution has been driven by strong selective pressure on S. It has been predicted that increasing immunity by natural infection or vaccination will increase the selective pressure to find a susceptible host, either by increased transmissibility or antibody evasion. It appears that Omicron has achieved both of these goals, although our data only speak directly to antibody evasion.

In addition to changes in the ACE2 footprint, Omicron RBD possesses a triplet of mutations from serines to more bulky, hydrophobic residues, a motif not found in any other Sarbecoviruses. This introduces structural changes and may lead to loss of the ability to form the lipid-binding pocket, which might normally aid release of the virus from infected cells. One of these mutations requires a double change in the codon, reinforcing its significance, and it is conceivable that there is synergy with the change at residue 498, perhaps explaining why this mutation has not established itself earlier.

For most mAbs, the changes in interaction are so severe that the activity is completely lost or greatly impaired. This also extends to the set of mAbs developed for clinical use—the activity of most is lost, AZD8895 and ADG20 activity is substantially reduced, whereas the activity of Vir S309 is more modestly reduced.

Omicron has now got a foothold in many countries. In the UK, it has an estimated doubling time of 2.5 days and 2 doses of vaccine appear to give low protection from infection, whereas 3 doses give better protection. There is considerable concern that Omicron will rapidly replace Delta and cause a large and sharp peak of infection in early 2022. It is likely that substantial increases in transmissibility and immune evasion are contributing to the explosive rise in Omicron infections. At present, the only option to control the spread of Omicron, barring social distancing and mask wearing, is to pursue vaccination with Wuhan-containing antigen to boost the response to sufficiently high titers to provide some protection. However, the antigenic distance of Omicron may mandate the development of vaccines against this strain. There will then be a question of how to use these vaccines; vaccination with Omicron will likely give good protection against Omicron but will not give good protection against other strains. Therefore, it seems possible that Omicron may cause a shift from the current monovalent vaccines containing Wuhan S to multivalent vaccines containing an antigen, such as Wuhan or Alpha, at the center of the antigenic map and Omicron or other S genes at the extreme peripheries of the map, similar to the polyvalent strategies used in influenza vaccines.

In summary, we have presented data showing that the huge number of mutational changes present in Omicron lead to a substantial knockdown of neutralizing capacity of immune serum and failure of mAb. This appears to lead to a fall in vaccine effectiveness, but it is unlikely that vaccines will completely fail and it is hoped that although vaccine breakthroughs will occur, protection from severe disease will be maintained, perhaps by T cells. It is likely that the vaccine-induced T cell response to SARS-CoV-2 will be less affected than the antibody response. Third-dose vaccine boosters substantially raise neutralization titers to Omicron and are the mainstay of the response to Omicron in countries, such as the UK. Widespread vaccine breakthroughs may mandate the production of a vaccine tailored to Omicron, and failure of mAbs may likewise lead to the generation of second-generation mAbs targeting Omicron.

A question asked after the appearance of each new variant is whether SARS-CoV-2 has reached its limit for evolution. Analyzing the mutations in Omicron shows that, except for S371L, all other mutations require only single-nucleotide changes. Two-nucleotide mutations and epistatic mutations are more difficult to reach, but they open up vast untapped potential for future variants. Global control measures are critical to avoid this.

### Limitations

The neutralization assays presented in this paper are performed *in vitro* and do not fully quantify the antibody response *in vivo*, where complement and antibody-dependent cell-mediated cytotoxicity may contribute to virus control. Evasion of the antibody response may allow reinfection with Omicron, but the role of the T cell response, which is not measured here, is likely to contribute to the control of infection and disease severity.

## STAR★Methods

### Key resources table

**Table:** 

REAGENT or RESOURCE	SOURCE	IDENTIFIER
**Antibodies**		

Fab	(Dejnirattisai et al., 2021a)	N/A
IgG	(Dejnirattisai et al., 2021a, Liu et al., 2021b)	N/A
Human anti-NP (mAb 206)	(Dejnirattisai et al., 2021a)	N/A
EY6A mAb	Zhou et al., 2020	N/A
Regeneron mAbs	AstraZeneca	Cat#REGN10933, and REGN10987
AstraZeneca mAbs	AstraZeneca	Cat#AZD1061, AZD8895
Vir mAbs	Adagio	Cat#S309
Lilly mAbs	Adagio	Cat#Ly-CoV555, and Cat#Ly-CoV16
Adagio mAbs	Adagio	Cat#ADG10, Cat#ADG20, and Cat#ADG30
Anti-Human IgG (Fc specific)-Peroxidase	Sigma	Cat#A0170; RRID:AB_257868
Polyclonal Rabbit Anti-Goat Immunoglobulins/FITC	DAKO	Cat#F0250
Anti-c-Myc 9E10 antibody	Biolegend	Catt#626872; RRID; AB_626872
Anti-mouse IgG(Fc specific)-FITC antibody	Merck/Sigma Aldrich	Catt#F4143; RRID:AB_259587

**Bacterial and virus strains and yeast**		

SARS-CoV-2 (Australia/VIC01/2020)	Caly et al., 2020	N/A
SARS-CoV-2/Alpha	Public Health England	N/A
SARS-CoV-2/Beta	Public Health England	N/A
SARS-CoV-2/Gamma	Dejnirattisai et al., 2021	N/A
SARS-CoV-2/Delta	W. Barclay	Imperial College London
SARS-CoV-2/Omicron	This paper	N/A
DH5α bacteria	In Vitrogen	Cat#18263012
Saccharomyces cerevisiae EBY100	ATCC	Cat#MYA-4941
E. coli cloni 10G cells	Lucigen, USA	Cat#60117-1

**Biological samples**		

Serum from Pfizer-vaccinated individuals	University of Oxford	N/A
Serum from AstraZeneca-Oxford-vaccinated individuals	University of Oxford	N/A
Plasma from SARS-CoV-2 patients	John Radcliffe Hospital in Oxford UK, South Africa, and FIOCRUZ (WHO) Brazil	N/A

**Chemicals, peptides, and recombinant proteins**		

His-tagged SARS-CoV-2 RBD	(Dejnirattisai et al., 2021a)	N/A
His-tagged SARS-CoV-2/Omicron RBD	This paper	N/A
His-tagged SARS-CoV-2 RBD-62	Zahradnik et al., 2021b	N/A
His-tagged SARS-CoV-2 RBD N501Y	Supasa et al., 2021	N/A
His-tagged SARS-CoV-2 RBD K417N, E484K, N501Y	Zhou et al., 2021	N/A
His-tagged SARS-CoV-2 RBD K417T, E484K, N501Y	Dejnirattisai et al., 2021b	N/A
His-tagged SARS-CoV-2 RBD L452R, T478K	Liu et al., 2021a	N/A
His-tagged human ACE2	Liu et al., 2021a	N/A
Human ACE2-hIgG1Fc	Liu et al., 2021a	N/A
His-tagged 3C protease	Libby et al., 1988	N/A
Phosphate buffered saline tablets	Sigma-Aldrich	Cat#P4417
Dulbecco’s Modified Eagle Medium, high glucose	Sigma-Aldrich	Cat#D5796
Dulbecco’s Modified Eagle Medium, low glucose	Sigma-Aldrich	Cat#D6046
FreeStyle 293 Expression Medium	Gibco	Cat#12338018
L-Glutamine–Penicillin–Streptomycin solution	Sigma-Aldrich	Cat#G1146
GlutaMAX Supplement	Gibco	Cat#35050061
UltraDOMA PF Protein-free Medium	Lonza	Cat#12-727F
Opti-MEM	Gibco	Cat#11058021
Fetal Bovine Serum	Gibco	Cat#12676029
Polyethylenimine, branched	Sigma-Aldrich	Cat#408727
Carboxymethyl cellulose	Sigma	Cat#C4888
Strep-TactinXT	IBA Lifesciences	Cat#2-1206-025
HEPES	Melford	Cat#34587-39108
Sodium Chloride	Honeywell	Cat#SZBF3340H
LB broth	Fisher Scientific UK	Cat#51577-51656
Mem Neaa (100X)	Gibco	Cat#2203945
Trypsin-EDTA	Gibco	Cat#2259288
TrypLE Express Enzyme	Gibco	Cat#12604013
L-Glutamine 200 mM (100X)	Gibco	Cat#2036885
SYPROorange (5000X in DMSO)	Thermo	Cat#S6651
Isopropyl β-d-1-thiogalactopyranoside	Meridian Bioscience	Cat#BIO-37036
Kanamycin	Melford	Cat#K22000
Lysozyme	Sigma-Aldrich	Cat#L6876
Tris-base	Melford	Cat#T60040
Imidazole	Sigma-Aldrich	Cat#56750
Triton-X-100	Sigma-Aldrich	Cat#8787
Turbonuclease	Sigma-Aldrich	Cat#T4330
RNAse A	Qiagen	Cat#158922
NaCl	Sigma-Aldrich	Cat#S9888
MgSO4	Sigma-Aldrich	Cat#746452
Na2HPO4	Melford	Cat#S23100
NaH2PO4	Melford	Cat#S23185
SD-CAA media	Zahradnik et al., 2021a	N/A
CF640-ACE2	Zahradnik et al., 2021b	N/A
HBS-EP+ Buffer 10×	Cytiva	Cat# BR100669
Regeneration Solution (glycine-HCl pH 1.7)	Cytiva	Cat# BR100838

**Deposited data**		

Crystal structures of SARS-CoV-2 Omicron-RBD/Beta-55 and EY6A Fab complex	This paper	PDB:7QNW
Crystal structures of SARS-CoV-2 RBD/Beta-55 and EY6A Fab complex	This paper	PDB:7QNX
Crystal structures of SARS-CoV-2 RBD/COVOX-58 and COVOX-158 Fab complex	This paper	PDB:7QNY

**Experimental models: Cell lines**		

HEK293S GnTI- cells	ATCC	Cat#CRL-3022
HEK293 cells	ATCC	Cat#CRL-3216
Expi293F™ Cells	Gibco,	Cat#A14527
HEK293T/17 cells	ATCC	Cat#CRL-11268™
HEK293T cells	ATCC	Cat#CRL-11268
Hamster: ExpiCHO cells	Thermo Fisher	Cat#A29133
Vero CCL-81 cells	ATCC	Cat#CCL-81
VeroE6/TMPRSS2 cells	NIBSC	Ref. no. 100978

**Recombinant DNA**		

Vector: pHLsec	Aricescu et al., 2006	N/A
Vector: pNEO	Aricescu et al., 2006	N/A
Vector: pHLsec-SARS-CoV-2 spike of Omicron	This paper	N/A
Vector: pNEO-SARS-CoV-2 RBD of Omicron	This paper	N/A
Vector: pCMV-VSV-G	Stewart et al., 2003	Addgene plasmid # 8454
pHR-SIN-ACE2	Alain Townsend	N/A
Vector: pOPING-ET	Nettleship et al., 2008	N/A
Vector: human IgG1 heavy chain	German Cancer Research Center, Heidelberg, Germany (H. Wardemann	N/A
Vector: human lambda light chain	German Cancer Research Center, Heidelberg, Germany (H. Wardemann	N/A
Vector: human kappa light chain	German Cancer Research Center, Heidelberg, Germany (H. Wardemann	N/A
Vector: Human Fab	Univeristy of Oxford	N/A
Vector: pJYDC1	Adgene	ID: 162458

**Software and algorithms**		

COOT	(Emsley et al., 2010)	https://www2.mrc-lmb.cam.ac.uk/personal/pemsley/coot/
Xia2-dials	Winter et al., 2018	https://xia2.github.io/parameters.html
PHENIX	Liebschner et al., 2019	https://www.phenix-online.org/
PyMOL	(Schrödinger and DeLano, 2020)	https://pymol.org/
Data Acquisition Software 11.1.0.11	Fortebio	https://www.fortebio.com/products/octet-systems-software
Data Analysis Software HT 11.1.0.25	Fortebio	https://www.fortebio.com/products/octet-systems-software
Prism 8.0	GraphPad	https://www.graphpad.com/scientific-software/prism/
Yeast display titration curve fitting were done by the standard non-cooperative Hill equation, fitted by nonlinear least-squares regression with two additional parameters using Python 3.7	Zahradnik et al., 2021b	N/A
IBM SPSS Software 27	IBM	https://www.ibm.com
mabscape	This paper	https://github.com/helenginn/mabscape https://snapcraft.io/mabscape

**Other**		

X-ray data were collected at beamline I03, Diamond Light Source, under proposal ib27009 for COVID-19 rapid access	This paper	https://www.diamond.ac.uk/covid-19/for-scientists/rapid-access.html
TALON Superflow Metal Affinity Resin	Clontech	Cat#635668
HiLoad 16/600 Superdex 200 pg	Cytiva	Cat#28-9893-35
Superdex 200 increase 10/300 GL column	Cytiva	Cat#28990944
HisTrap nickel HP 5-ml column	Cytiva	Cat#17524802
HiTrap Heparin HT 5-ml column	Cytiva	Cat#17040703
Amine Reactive Second-Generation (AR2G) Biosensors	Fortebio	Cat#18-5092
Octet RED96e	Fortebio	https://www.fortebio.com/products/label-free-bli-detection/8-channel-octet-systems
Buffer exchange system “QuixStand”	GE Healthcare	Cat#56-4107-78
Cartesian dispensing system	Genomic solutions	Cat#MIC4000
Hydra-96	Robbins Scientific	Cat#Hydra-96
96-well crystallization plate	Greiner bio-one	Cat#E20113NN
Crystallization Imaging System	Formulatrix	Cat#RI-1000
Sonics vibra-cell vcx500 sonicator	VWR	Cat#432-0137

### Resource availability

#### Lead contact

Resources, reagents and further information requirement should be forwarded to and will be responded by the lead contact, David I Stuart (dave@strubi.ox.ac.uk).

#### Materials availability

Reagents generated in this study are available from the lead contact with a completed Materials Transfer Agreement.

### Experimental model and subject details

#### Viral stocks

SARS-CoV-2/, S371L SARS-CoV-2/human/AUS/VIC01/2020 (Caly et al., 2020), Alpha and Beta were provided by Public Health England, Gamma cultured from a throat swab from Brazil, Delta was a gift from Wendy Barclay and Thushan de Silva, from the UK G2P genotype to phenotype consortium and Omicron was grown from a positive throat swab (IRAS Project ID: 269573, Ethics Ref: 19/NW/0730. Briefly, VeroE6/TMPRSS2 cells (NIBSC) were maintained in Dulbecco's Modified Eagle Medium (DMEM) high glucose supplemented with 1% fetal bovine serum, 2mM Glutamax, 100 IU/ml penicillin-streptomycin and 2.5ug/ml amphotericin B, at 37 °C in the presence of 5% CO2 before inoculation with 200ul of swab fluid. Cells were further maintained at 37°C with daily observations for cytopathic effect (CPE). Virus-containing supernatants were clarified at 80% CPE by centrifugation at 3,000 r.p.m. at 4 °C before being stored at -80 °C in single-use aliquots. Viral titres were determined by a focus-forming assay on Vero CCL-81 cells (ATCC). Sequencing of the Omicron isolate shows the expected consensus S gene changes (A67V, Δ69-70, T95I, G142D/Δ143-145, Δ211/L212I, ins214EPE, G339D, S371L, S373P, S375F, K417N, N440K, G446S, S477N, T478K, E484A, Q493R, G496S, Q498R, N501Y, Y505H, T547K, D614G, H655Y, N679K, P681H, N764K, D796Y, N856K, Q954H, N969K, L981F), an intact furin cleavage site and a single additional mutation A701V. Cells were infected with the SARS-CoV-2 virus using an MOI of 0.0001.

Virus-containing supernatants were harvested at 80% CPE and spun at 3000 rpm at 4 °C before storage at -80 °C. Viral titres were determined by a focus-forming assay on Vero cells. Victoria passage 5, Alpha passage 2 and Beta passage 4 stocks Gamma passage 1, Delta passage 3 and Omicron passage 1 were sequenced to verify that they contained the expected spike protein sequence and no changes to the furin cleavage sites.

#### Bacterial strains and cell culture

Vero, Vero (ATCC CCL-81) and VeroE6/TMPRSS2 cells were cultured at 37 °C in Dulbecco’s Modified Eagle medium (DMEM) high glucose (Sigma-Aldrich) supplemented with 10% fetal bovine serum (FBS), 2 mM GlutaMAX (Gibco, 35050061) and 100 U/ml of penicillin–streptomycin. Human mAbs were expressed in HEK293T cells cultured in UltraDOMA PF Protein-free Medium (Cat#12-727F, LONZA) at 37 °C with 5 % CO_2_. HEK293T (ATCCCRL -11268) cells were cultured in DMEM high glucose (Sigma-Aldrich) supplemented with 10 % FBS, 1 % 100X Mem Neaa (Gibco) and 1 %100X L-Glutamine (Gibco) at 37 °C with 5 % CO_2_. To express RBD, RBD variants and ACE2, HEK293T cells were cultured in DMEM high glucose (Sigma) supplemented with 2 % FBS, 1 % 100X Mem Neaa and 1 % 100X L-Glutamine at 37 °C for transfection. Omicron RBD and human mAbs were also expressed in HEK293T (ATCCCRL-11268) cells cultured in FreeStyle 293 Expression Medium (ThermoFisher,12338018) at 37 °C with 5 % CO_2_. E .coli DH5α bacteria were used for transformation and large-scalepreparation of plasmids. A single colony was picked and cultured in LB broth at 37 °C at 200 rpm in a shaker overnight.

#### Plasma from early pandemic and Alpha cases

Participants from the first wave of SARS-CoV2 in the U.K. and those sequence confirmed with B.1.1.7 lineage in December 2020 and February 2021 were recruited through three studies: Sepsis Immunomics [Oxford REC C, reference:19/SC/0296]), ISARIC/WHO Clinical Characterisation Protocol for Severe Emerging Infections [Oxford REC C, reference 13/SC/0149] and the Gastro-intestinal illness in Oxford: COVID sub study [Sheffield REC, reference: 16/YH/0247]. Diagnosis was confirmed through reporting of symptoms consistent with COVID-19 and a test positive for SARS-CoV-2 using reverse transcriptase polymerase chain reaction (RT-PCR) from an upper respiratory tract (nose/throat) swab tested in accredited laboratories. A blood sample was taken following consent at least 14 days after symptom onset. Clinical information including severity of disease (mild, severe or critical infection according to recommendations from the World Health Organisation) and times between symptom onset and sampling and age of participant was captured for all individuals at the time of sampling. Following heat inactivation of plasma/serum samples they were aliquoted so that no more than 3 freeze thaw cycles were performed for data generation.

#### Sera from Beta-, Gamma-, and Delta-infected cases

Beta and Delta samples from UK infected cases were collected under the “Innate and adaptive immunity against SARS-CoV-2 in healthcare worker family and household members” protocol affiliated to the Gastro-intestinal illness in Oxford: COVID sub study discussed above and approved by the University of Oxford Central University Research Ethics Committee. All individuals had sequence confirmed Beta/Delta infection or PCR-confirmed symptomatic disease occurring whilst in isolation and in direct contact with Beta/Delta sequence-confirmed cases. Additional Beta infected serum (sequence confirmed) was obtained from South Africa. At the time of swab collection patients signed an informed consent to consent for the collection of data and serial blood samples. The study was approved by the Human Research Ethics Committee of the University of the Witwatersrand (reference number 200313) and conducted in accordance with Good Clinical Practice guidelines. Gamma samples were provided by the International Reference Laboratory for Coronavirus at FIOCRUZ (WHO) as part of the national surveillance for coronavirus and had the approval of the FIOCRUZ ethical committee (CEP 4.128.241) to continuously receive and analyse samples of COVID-19 suspected cases for virological surveillance. Clinical samples were shared with Oxford University, UK under the MTA IOC FIOCRUZ 21-02.

#### Sera from Pfizer vaccinees

Pfizer vaccine serum was obtained from volunteers who had received two and three doses of the BNT162b2 vaccine. Vaccinees were Health Care Workers, based at Oxford University Hospitals NHS Foundation Trust, not known to have prior infection with SARS-CoV-2 and were enrolled in the OPTIC Study as part of the Oxford Translational Gastrointestinal Unit GI Biobank Study 16/YH/0247 [research ethics committee (REC) at Yorkshire & The Humber – Sheffield] which has been amended for this purpose on 8 June 2020. The study was conducted according to the principles of the Declaration of Helsinki (2008) and the International Conference on Harmonization (ICH) Good Clinical Practice (GCP) guidelines. Written informed consent was obtained for all participants enrolled in the study. Participants were sampled approximately 28 days (range 25-38), 180 days (range 178-221) and 270 days (range 243-273) after receiving two doses of Pfizer/BioNtech BNT162b2 mRNA Vaccine, 30 micrograms, administered intramuscularly after dilution (0.3 mL each), 17-28 days apart, then approximately 28 days (range 25-56) after receiving a third "booster" dose of BNT162B2 vaccine. The mean age of vaccinees was 42 years (range 30-59), 10 male and 10 female.

#### AstraZeneca-Oxford vaccine study procedures and sample processing

Full details of the randomized controlled trial of ChAdOx1 nCoV-19 (AZD1222), were previously published (PMID: 33220855/PMID: 32702298). These studies were registered at ISRCTN (15281137 and 89951424) and ClinicalTrials.gov (NCT04324606 and NCT04400838). Written informed consent was obtained from all participants, and the trial is being done in accordance with the principles of the Declaration of Helsinki and Good Clinical Practice. The studies were sponsored by the University of Oxford (Oxford, UK) and approval obtained from a national ethics committee (South Central Berkshire Research Ethics Committee, reference 20/SC/0145 and 20/SC/0179) and a regulatory agency in the United Kingdom (the Medicines and Healthcare Products Regulatory Agency). An independent DSMB reviewed all interim safety reports. A copy of the protocols was included in previous publications (Folegatti et al., 2020).

Data from vaccinated volunteers who received three vaccinations are included in this paper. Vaccine doses were either 5 × 10^10^ viral particles (standard dose; SD/SD cohort n=21) or half dose as their first dose (low dose) and a standard dose as their second dose (LD/SD cohort n=4). The interval between first and second dose was in the range of 8-14 weeks. Blood samples were collected and serum separated on the day of vaccination and on pre-specified days after vaccination e.g. 14 and 28 days after boost.

### Method details

#### Focus Reduction Neutralization Assay (FRNT)

The neutralization potential of Ab was measured using a Focus Reduction Neutralization Test (FRNT), where the reduction in the number of the infected foci is compared to a negative control well without antibody. Briefly, serially diluted Ab or plasma was mixed with SARS-CoV-2 strains for 1 hr at 37 °C. The mixtures were then transferred to 96**-**well**,** cell culture-treated, flat-bottom microplates containing confluent Vero cell monolayers in duplicate and incubated for a further 2 hrs followed by the addition of 1.5% semi-solid carboxymethyl cellulose (CMC) overlay medium to each well to limit virus diffusion. A focus forming assay was then performed by staining Vero cells with human anti-NP mAb (mAb206) followed by peroxidase-conjugated goat anti-human IgG (A0170; Sigma). Finally, the foci (infected cells) approximately 100 per well in the absence of antibodies, were visualized by adding TrueBlue Peroxidase Substrate. Virus-infected cell foci were counted on the classic AID EliSpot reader using AID ELISpot software. The percentage of focus reduction was calculated and IC_50_ was determined using the probit program from the SPSS package.

#### DNA manipulations

Cloning was done by using a restriction-free approach (Peleg and Unger, 2014). Mutagenic megaprimers were PCR amplified (KAPA HiFi HotStart ReadyMix, Roche, Switzerland, cat. KK3605), purified by using NucleoSpin® Gel and PCR Clean-up kit (Nacherey-Nagel, Germany, REF 740609.50) and cloned into pJYDC1 (Adgene ID: 162458) (Zahradnik et al., 2021a). Parental pJYDC1 molecules were cleaved by DpnI treatment (1 h, NEB, USA, cat. R0176) and the reaction mixture was electroporated into E.coli Cloni® 10G cells (Lucigen, USA). The correctness of mutagenesis was verified by sequencing.

#### Yeast display binding assays

Plasmids (pJYDC1) with mutations were transformed (1 ug of DNA) by LiAc method (Gietz and Woods, 2006) into the EBY100 Saccharomyces cerevisiae and selected by growth on SD-W plates (Zahradnik et al., 2021a) for 48-72 h at 30°C. Grown single colonies were transferred to 1.0 ml liquid SD-CAA media, grown 24 h or 48 (RBD-Omicron) at 30°C (220 rpm), and 50 ul of the starter culture was used as inoculum (5 %) for the expression culture in 1/9 media (1 ml) supplemented with 1 nM DMSO solubilized bilirubin (Merck/Sigma-Aldrich cat. B4126). The expression continued in a shaking incubator for 24 h at 20°C (220 rpm). Aliquots of yeast expressed cells (100 ul, 3000 g, 3 min) were washed in ice-cold PBSB buffer (PBS with 1 g/L BSA) and resuspended in PBSB with a dilution series CF640-ACE2 (1 pM – 80 nM). The volume and incubation times were adjusted to limit the ligand depletion effect and enable equilibrium (Zahradnik et al., 2021b). After incubation, cells were washed in ice-cold PBSB buffer (PBS with 1 g/L BSA) passed through cell strainer nylon membrane (40 μM, SPL Life Sciences, Korea), and analyzed. The yeast expressing Omicron-RBD were expression labelled by primary anti-c-Myc 9E10 antibody (Biolegend, Cat. 626872) and secondary Anti-Mouse IgG(Fc specific)-FITC (Merck/Sigma-Aldrich, cat. F4143) antibodies. The signals for expression (FL1, eUnaG2 fluorophore, Ex. 498 nm, Em. 527 nm or FITC) and for binding (FL3, CF®640R dye-labeled ACE2) were recorded by S3e Cell Sorter (BioRad, USA). The standard non-cooperative Hill equation was fitted by nonlinear least-squares regression with two additional parameters using Python 3.7 (Starr et al., 2020; Zahradnik et al., 2021a).

#### Antigenic mapping

Antigenic mapping of Omicron was carried out through an extension of a previous algorithm (Liu et al., 2021a). In short, coronavirus variants were assigned three-dimensional coordinates whereby the distance between two points indicates the base drop in neutralization titre. Each serum was assigned a strength parameter which provided a scalar offset to the logarithm of the neutralization titre. These parameters were refined to match predicted neutralization titres to observed values by taking an average of superimposed positions from 30 separate runs. The three-dimensional positions of the variants of concern: Victoria, Alpha, Beta, Gamma, Delta and Omicron were plotted for display.

#### Alphafold

Models of Omicron RBD and NTD were derived using AlphaFold 2.0.01 (Jumper et al., 2021) downloaded and installed on 11^th^ August 2021 in batch mode. For RBD predictions, 204 residues (P327-N529) were used as an input sequence while the NTD sequence input was from residues V1-S253. The max_release_date parameter was set to 28-11-2021 when the simulations were run such that template information was used for structure modelling. For all targets, five models were generated and all presets were kept the same.

#### Cloning of Spike and RBD

Expression plasmids of wild-type and Omicron spike and RBD were constructed encoding for human codon-optimized sequences from wild-type SARS-CoV-2 (MN908947) and Omicron (EPI_ISL_6640917). Wild-type Spike and RBD plasmids were constructed as described before (Dejnirattisai et al., 2021a). Spike and RBD fragments of Omicron were custom synthesized by GeneArt (Thermo Fisher Scientific GENEART) and cloned into pHLsec and pNEO vectors, respectively, as previously described (Dejnirattisai et al., 2021a; Supasa et al., 2021; Zhou et al., 2021). Both constructs were verified by Sanger sequencing after plasmid isolation using QIAGEN Miniprep kit (QIAGEN).

#### Protein production

Protein expression and purification were conducted as described previously (Dejnirattisai et al., 2021a; Zhou et al., 2020). Briefly, plasmids encoding proteins were transiently expressed in HEK293T (ATCC CRL-11268) cells. The conditioned medium was concentrated using a QuixStand benchtop system. His-tagged Omicron RBD were purified with a 5 mL HisTrap nickel column (GE Healthcare) and further polished using a Superdex 75 HiLoad 16/60 gel filtration column (GE Healthcare). Twin-strep tagged Omicron spike was purified with Strep-Tactin XT resin (IBA lifesciences).

∼4mg of ACE2 was mixed with homemade His-tagged 3C protease and DTT (final concentration 1mM). After incubation at 4 °C for one day, the sample was flowed through a 5 mL HisTrap nickel column (GE Healthcare). His-tagged proteins were removed by the nickel column and purified ACE2 was harvested and concentrated.

#### IgG mAbs and Fab purification

To purify full length IgG mAbs, supernatants of mAb expression were collected and filtered by a vacuum filter system and loaded on protein A/G beads over night at 4 °C. Beads were washed with PBS three times and 0.1 M glycine pH 2.7 was used to elute IgG. The eluate was neutralized with Tris-HCl pH 8 buffer to make the final pH=7. The IgG concentration was determined by spectrophotometry and buffer exchanged into PBS.

To express and purify Fabs 158 and EY6A, heavy chain and light chain expression plasmids of Fab were co-transfected into HEK293T cells by PEI. After culturing cells for 5 days at 37°C with 5% CO2, culture supernatant was harvested and filtered using a 0.22 mm polyethersulfone filter. Fab 158 was purified using Strep-Tactin XT resin (IBA lifesciences) and Fab EY6A was purified with Ni-NTA column (GE HealthCare) and a Superdex 75 HiLoad 16/60 gel filtration column (GE Healthcare).

AstraZeneca and Regeneron antibodies were provided by AstraZeneca, Vir, Lilly and Adagio antibodies were provided by Adagio. For the antibodies heavy and light chains of the indicated antibodies were transiently transfected into 293Y cells and antibody purified from supernatant on protein A. Fab fragments of 58 and beta-55 were digested from purified IgGs with papain using a Pierce Fab Preparation Kit (Thermo Fisher), following the manufacturer’s protocol.

#### Surface Plasmon Resonance

The surface plasmon resonance experiments were performed using a Biacore T200 (GE Healthcare). All assays were performed with a running buffer of HBS-EP (Cytiva) at 25°C.

To determine the binding kinetics between the SARS-CoV-2 RBDs and ACE2 / monoclonal antibody (mAb), a Protein A sensor chip (Cytiva) was used. ACE2-Fc or mAb was immobilised onto the sample flow cell of the sensor chip. The reference flow cell was left blank. RBD was injected over the two flow cells at a range of five concentrations prepared by serial twofold dilutions, at a flow rate of 30μl min^−1^ using a single-cycle kinetics programme. Running buffer was also injected using the same programme for background subtraction. All data were fitted to a 1:1 binding model using Biacore T200 Evaluation Software 3.1.

#### Crystallization

Wuhan RBD was mixed with mAb-58 and mAb-158 Fabs, Wuhan or Omicron RBD was mixed with EY6A and beta-55 Fabs, in a 1:1:1 molar ratio, with a final concentration of 7, 7 and 3 mg ml^−1^. These complexes were separately incubated at room temperature for 30 min. Initial screening of crystals was set up in Crystalquick 96-well X plates (Greiner Bio-One) with a Cartesian Robot using the nanoliter sitting-drop vapor-diffusion method, with 100 nL of protein plus 100 nL of reservoir in each drop for Wuhan RBD/mAb-58/mAb-158 and Wuhan RBD/EY6A/beta-55 complexes, and 200 nL of protein plus 100 nL of reservoir for Omicron RBD/EY6A/beta-55 complex, as previously described (Walter et al., 2003). Crystals of Wuhan RBD/mAb-58/mAb-158 were formed in Hampton Research PEGRx condition 2-28, containing 0.1 M sodium citrate tribasic, pH 5.5 and 20% (w/v) PEG 4000. Crystals of Wuhan RBD/EY6A/beta-55 complex were obtained from Emerald Biostructures Wizard condition II-7, containing 0.2 M NaCl, 0.1 M Tris, pH 6.7 and 30% (w/v) PEG 3000. Crystals of Omicron RBD/EY6A/beta-55 complex were formed in Hampton Research Index condition 80, containing 0.2 M (NH4)2COOH, 0.1 M Hepes, pH 7.5 and 25% (w/v) PEG 3350.

#### X-ray data collection, structure determination, and refinement

Crystals were mounted in loops and dipped in solution containing 25% glycerol and 75% mother liquor for a second before being frozen in liquid nitrogen. Diffraction data were collected at 100 K at beamline I03 of Diamond Light Source, UK. All data were collected as part of an automated queue system allowing unattended automated data collection (https://www.diamond.ac.uk/Instruments/Mx/I03/I03-Manual/Unattended-Data-Collections.html). Diffraction images of 0.1° rotation were recorded on an Eiger2 XE 16M detector (exposure time from 0.02 to 0.03 s per image, beam size 80×20 μm or 50×20 μm, 10% to 30% beam transmission and wavelength of 0.9763 Å). Data were indexed, integrated and scaled with the automated data processing program Xia2-dials (Winter, 2010, Winter et al., 2018). 720° of data were collected from a crystal of Omicron-RBD/Beta-55/EY6A. 360° of data were collected for each of the Wuhan RBD/Beta-55/EY6A and Wuhan RDB/mAb-58/mAb-158 data sets.

Structures were determined by molecular replacement with PHASER (McCoy et al., 2007). VhVl and ChCl domains which have the most sequence similarity to previously determined SARS-CoV-2 RBD/Fab structures (Dejnirattisai et al., 2021a, 2021b; Huo et al., 2020; Liu et al., 2021a; Supasa et al., 2021; Zhou et al., 2020, 2021) were used as search models for each of the current structure determination. Model rebuilding with COOT (Emsley et al., 2010) and refinement with Phenix (Liebschner et al., 2019) were used for all the structures. Data collection and structure refinement statistics are given in Table S2. Structural comparisons used SHP (Stuart et al., 1979), residues forming the RBD/Fab interface were identified with PISA (Krissinel and Henrick, 2007) and figures were prepared with PyMOL (The PyMOL Molecular Graphics System, Version 1.2r3pre, Schrödinger, LLC).

### Quantification and statistical analysis

Statistical analyses are reported in the results and figure legends. Neutralization was measured by FRNT. The percentage of focus reduction was calculated and IC50 (FRNT50) was determined using the probit program from the SPSS package. The Wilcoxon matched-pairs signed rank test was used for the analysis and two-tailed P values were calculated on geometric mean values.

## Data Availability

The coordinates and structure factors of the crystallographic complexes are available from the PDB with accession codes given in Table S2. Mabscape is available from https://github.com/helenginn/mabscape, https://snapcraft.io/mabscape. The data that support the findings of this study are available from the corresponding authors on request.
