# Supplementary material for: SARS-CoV-2 Omicron-B.1.1.529 leads to widespread escape from neutralizing antibody responses
Source: Cell. 2022 Feb 3;185(3):467–484.e15. doi: 10.1016/j.cell.2021.12.046 (PMC8723827; doi:10.1016/j.cell.2021.12.046)
Supplement: Document S1. Tables S1 and S2 [file mmc1.pdf]

Cell, Volume *185*

Supplemental information

SARS-CoV-2 Omicron-B.1.1.529 leads to widespread escape from neutralizing antibody responses

Wanwisa Dejnirattisai, Jiandong Huo, Daming Zhou, Jiří Zahradník, Piyada Supasa, Chang Liu, Helen M.E. Duyvesteyn, Helen M. Ginn, Alexander J. Mentzer, Aekkachai Tuekprakhon, Rungtiwa Nutalai, Beibei Wang, Aiste Dijokaite, Suman Khan, Ori Avinoam, Mohammad Bahar, Donal Skelly, Sandra Adele, Sile Ann Johnson, Ali Amini, Thomas G. Ritter, Chris Mason, Christina Dold, Daniel Pan, Sara Assadi, Adam Bellass, Nicola Omo-Dare, David Koeckerling, Amy Flaxman, Daniel Jenkin, Parvinder K. Aley, Merryn Voysey, Sue Ann Costa Clemens, Felipe Gomes Naveca, Valdinete Nascimento, Fernanda Nascimento, Cristiano Fernandes da Costa, Paola Cristina Resende, Alex Pauvolid-Correa, Marilda M. Siqueira, Vicky Baillie, Natali Serafin, Gaurav Kwatra, Kelly Da Silva, Shabir A. Madhi, Marta C. Nunes, Tariq Malik, Peter J.M. Openshaw, J. Kenneth Baillie, Malcolm G. Semple, Alain R. Townsend, Kuan-Ying A. Huang, Tiong Kit Tan, Miles W. Carroll, Paul Klenerman, Eleanor Barnes, Susanna J. Dunachie, Bede Constantinides, Hermione Webster, Derrick Crook, Andrew J. Pollard, Teresa Lambe, OPTIC Consortium, ISARIC4C Consortium, Neil G. Paterson, Mark A. Williams, David R. Hall, Elizabeth E. Fry, Juthathip Mongkolsapaya, Jingshan Ren, Gideon Schreiber, David I. Stuart, and Gavin R. Screaton

mAb	IC50 (ug/ml)					
	Victoria	Alpha	Beta	Gamma	Delta	Omicron
40	0.026 ± 0.007	0.035 ± 0.008	0.738 ± 0.311	0.153 ± 0.037	0.029 ± 0.010	7.989±2.011
55	0.095 ± 0.015	0.348 ± 0.044	0.127 ± 0.014	0.306 ± 0.046	0.016 ± 0.005	7.12±2.880
58	0.041 ± 0.003	0.116 ± 0.029	0.136 ± 0.010	0.236 ± 0.075	6.434 ± 2.623	0.141±0.063
88	0.033 ± 0.001	0.058 ± 0.008	>10	>10	0.039 ± 0.007	>10
132	0.048 ± 0.000	0.337 ± 0.048	>10	>10	0.051 ± 0.013	>10
150	0.012 ± 0.000	0.139 ± 0.019	0.350 ± 0.010	0.040 ± 0.003	0.020 ± 0.001	>10
158	0.031 ± 0.004	0.254 ± 0.109	>10	>10	0.026 ± 0.002	>10
159	0.011 ± 0.000	0.061 ± 0.020	>10	1.434 ± 0.804	>10	>10
165	0.034 ± 0.004	0.212 ± 0.004	0.054 ± 0.013	0.241 ± 0.030	0.027 ± 0.006	>10
170	0.025 ± 0.004	0.105 ± 0.050	>10	>10	0.841 ± 0.103	>10
175	0.026 ± 0.000	0.575 ± 0.280	>10	3.881 ± 0.738	0.017 ± 0.003	>10
222	0.019 ± 0.000	0.014 ± 0.002	0.017 ± 0.005	0.008 ± 0.003	0.018 ± 0.001	0.240±0.122
253	0.055 ± 0.008	0.126 ± 0.018	0.109 ± 0.055	0.137 ± 0.005	0.005 ± 0.001	1.063±0.367
269	0.030 ± 0.000	>10	>10	>10	0.021 ± 0.004	>10
278	0.014 ± 0.007	0.307 ± 0.149	0.160 ± 0.018	0.245 ± 0.042	7.374 ± 1.397	>10
281	0.005 ± 0.001	0.012 ± 0.000	>10	>10	1.494 ± 0.302	>10
316	0.018 ± 0.007	0.024 ± 0.005	>10	>10	0.008 ± 0.001	>10
318	0.029 ± 0.008	0.185 ± 0.037	0.019 ± 0.008	0.083 ± 0.032	0.018 ± 0.003	>10
384	0.004 ± 0.001	0.005 ± 0.002	>10	>10	0.108 ± 0.035	>10
398	0.091 ± 0.004	0.180 ± 0.001	>10	>10	0.237 ± 0.038	>10
253-55	0.003 ± 0.000	0.008 ± 0.002	0.009 ± 0.002	0.026 ± 0.006	0.003 ± 0.000	2.945±1.283
253-165	0.003 ± 0.000	0.006 ± 0.000	0.013 ± 0.003	0.019 ± 0.000	0.007 ± 0.002	>10
β06	>10	0.024 ± 0.002	0.008 ± 0.002	0.015 ± 0.003	>10	>10
β10	>10	0.064 ± 0.042	0.015 ± 0.000	0.025 ± 0.011	>10	>10
β20	>10	>10	0.005 ± 0.001	0.345 ± 0.122	>10	7.518±1.105
β22	>10	6.58 ± 2.988	0.025 ± 0.004	0.030 ± 0.007	>10	0.393±0.234
β23	>10	0.009 ± 0.001	0.011 ± 0.001	0.020± 0.000	>10	>10
β24	>10	0.007 ± 0.001	0.002 ± 0.001	0.005 ± 0.001	>10	>10
β26	2.742 ± 0.208	>10	0.012 ± 0.003	0.016 ± 0.000	>10	>10
β27	0.018 ± 0.002	0.018 ± 0.000	0.009 ± 0.000	0.006 ± 0.002	0.021 ± 0.004	2.693±0.741
β29	>10	1.372 ± 0.016	0.027 ± 0.003	0.023 ± 0.009	>10	0.261±0.079
β30	2.643 ± 0.88	0.004 ± 0.001	0.003 ± 0.001	0.004 ± 0.000	0.350 ± 0.035	>10
β32	0.248 ± 0.003	0.119 ± 0.044	0.053 ± 0.025	0.027 ± 0.014	0.267 ± 0.068	>10
β33	2.016 ± 0.051	0.234 ± 0.013	0.017 ± 0.003	0.017 ± 0.001	0.334 ± 0.005	>10
β34	8.241 ± 1.067	1.466 ± 0.136	0.032 ± 0.010	0.092 ± 0.003	>10	>10
β38	>10	>10	0.011 ± 0.003	0.043 ± 0.025	>10	>10
β40	0.075 ± 0.005	0.001 ± 0.000	0.001 ± 0.000	0.001 ± 0.000	0.107 ± 0.031	0.012±0.007
β43	>10	>10	0.048±0.024	>10	>10	>10
β44	0.007 ± 0.002	0.028 ± 0.008	0.015 ± 0.008	0.071 ± 0.026	>10	>10
β45	>10	>10	0.018 ± 0.003	0.015 ± 0.006	>10	9.947±0.053
β47	0.006 ± 0.001	0.008 ± 0.003	0.003 ± 0.001	0.004 ± 0.002	0.005 ± 0.000	0.096±0.011
β48	0.034 ± 0.011	0.020 ± 0.008	0.009 ± 0.001	0.011 ± 0.001	0.042 ± 0.016	8.362±1.638
β49	0.009 ± 0.000	0.011 ± 0.001	0.007 ± 0.000	0.019 ± 0.003	0.008 ± 0.003	>10
β50	0.011 ± 0.000	0.014 ± 0.006	0.007 ± 0.001	0.015 ± 0.005	0.019 ± 0.005	>10
β51	0.119 ± 0.008	0.242 ± 0.024	0.005 ± 0.000	0.019 ± 0.001	>10	>10
β53	0.005 ± 0.000	0.032 ± 0.009	0.004 ± 0.000	0.017 ± 0.001	0.007 ± 0.000	0.266±0.117
β54	0.232 ± 0.092	0.002 ± 0.001	0.001 ± 0.000	0.002 ± 0.001	0.409 ± 0.071	0.020±0.011
β55	0.108 ± 0.069	0.028 ± 0.004	0.01 0± 0.003	0.022 ± 0.001	0.076 ± 0.020	0.109±0.013
β56	0.046 ± 0.013	0.001 ± 0.000	0.001 ± 0.000	0.002 ± 0.000	0.022 ± 0.002	0.194±0.056
AZD1061	0.013 ± 0.003	0.012 ± 0.002	0.014 ± 0.002	0.007 ± 0.002	0.038 ± 0.006	3.488±2.085
AZD8895	0.005 ± 0.001	0.011 ± 0.002	0.046 ± 0.031	0.046 ± 0.016	0.003 ± 0.000	1.152±0.170
AZD7442	0.009 ± 0.000	0.007 ± 0.001	0.012 ± 0.001	0.006 ± 0.003	0.005 ± 0.000	0.273±0.062
REGN10987	0.032 ± 0.007	0.028 ± 0.003	0.007 ± 0.001	0.013 ± 0.002	0.017 ± 0.009	>10
REGN10933	0.004 ± 0.002	0.014 ± 0.002	3.284 ± 2.014	6.177 ± 1.914	0.003 ± 0.001	>10
ADG10	0.006 ± 0.000	0.010 ± 0.001	0.011 ± 0.001	0.003 ± 0.000	0.026 ± 0.005	>10
ADG20	0.004 ± 0.001	0.006 ± 0.000	0.01 ± 0.001	0.009 ± 0.000	0.006 ± 0.001	1.104±0.509
ADG30	0.007 ± 0.002	0.016 ± 0.001	0.029 ± 0.003	0.002 ± 0.001	0.033 ± 0.007	>10
Ly-CoV-555	0.006 ± 0.002	0.009 ± 0.000	>10	>10	8.311 ± 4.059	>10
Ly-CoV16	0.034 ± 0.007	3.225 ± 1.030	>10	>10	0.012 ± 0.002	>10
S309	0.040 ± 0.005	0.078 ± 0.069	0.082 ± 0.002	0.076 ± 0.014	0.113 ± 0.028	0.256±0.034

Table S1. FRNT50 data, related to Figure 4.

**Structure**	**RBD/58-158** PDB:7QNY	**RBD/Beta-55-EY6A** PDB:7QNX	**Omicron-RBD/ Beta-55-EY6A** PDB:7QNW
**Data collection**			
Space group	$P3_121$	$P3_221$	$P3_221$
Cell dimensions			
a, *b*, *c* (Å)	72.8, 72.8, 416.2	131.8, 131.8, 116.8	132.0, 132.0, 117.3
α, β, γ (°)	90, 90, 120	90, 90, 120	90, 90, 120
Resolution (Å)	104–2.84 (2.89–2.84)	82–2.92 (2.97–2.92)	117–2.40 (2.44–2.40)
R_{merge}	0.290 (---)	0.554 (---)	0.339 (---)
R_{pim}	0.071 (0.737)	0.125 (0.902)	0.053 (0.458)
$I/\sigma(I)$	5.0 (0.3)	3.8 (0.5)	8.5 (0.7)
$CC_{1/2}$	0.996 (0.285)	0.986 (0.311)	0.977 (0.319)
Completeness (%)	100 (100)	99.9 (96.5)	100 (100)
Redundancy	17.9 (19.3)	20.5 (20.2)	41.1 (42.0)
**Refinement**			
Resolution (Å)	70–2.84	82–2.92	114–2.40
No. reflections	29939/1578	24493/1236	44201/2278
R_{work} / R_{free}	0.226/0.278	0.228/0.266	0.206/0.247
No. atoms			
Protein	7933	8097	8126
Ligand/ion/water	36	28	193
B factors ($Å^2$)			
Protein	116	74	65
Ligand/ion/water	114	119	70
r.m.s. deviations			
Bond lengths (Å)	0.002	0.002	0.002
Bond angles (°)	0.5	0.5	0.5

Values in parentheses are for highest-resolution shell.

Table S2 X-ray data collection and structure refinement statistics. Related to Figure 7.
